# Congolese Rhizospheric Soils as a Rich Source of New Plant Growth-Promoting Endophytic *Piriformospora* Isolates

**DOI:** 10.3389/fmicb.2017.00212

**Published:** 2017-02-14

**Authors:** Jolien Venneman, Kris Audenaert, Jan Verwaeren, Geert Baert, Pascal Boeckx, Adrien M. Moango, Benoît D. Dhed’a, Danny Vereecke, Geert Haesaert

**Affiliations:** ^1^Department of Applied Biosciences, Ghent UniversityGhent, Belgium; ^2^Isotope Bioscience Laboratory-ISOFYS, Ghent UniversityGhent, Belgium; ^3^Faculty of Science and Agriculture, Kisangani UniversityKisangani, Congo

**Keywords:** integrated soil fertility management, plant growth-promoting tropical endophytes, Sebacinales, species complex, *TEF1α* gene

## Abstract

In the last decade, there has been an increasing focus on the implementation of plant growth-promoting (PGP) organisms as a sustainable option to compensate for poor soil fertility conditions in developing countries. Trap systems were used in an effort to isolate PGP fungi from rhizospheric soil samples collected in the region around Kisangani in the Democratic Republic of Congo. With sudangrass as a host, a highly conducive environment was created for sebacinalean chlamydospore formation inside the plant roots resulting in a collection of 51 axenically cultured isolates of the elusive genus *Piriformospora* (recently transferred to the genus *Serendipita*). Based on morphological data, ISSR fingerprinting profiles and marker gene sequences, we propose that these isolates together with *Piriformospora williamsii* constitute a species complex designated *Piriformospora* (= *Serendipita*) ‘*williamsii.*’ A selection of isolates strongly promoted plant growth of *in vitro* inoculated *Arabidopsis* seedlings, which was evidenced by an increase in shoot fresh weight and a strong stimulation of lateral root formation. This isolate collection provides unprecedented opportunities for fundamental as well as translational research on the Serendipitaceae, a family of fungal endophytes in full expansion.

## Introduction

Worldwide, but particularly in developing countries, food security is threatened both by a growing population and by climate change. To meet the global demands, 70% more food will have to be produced by 2050, which can be achieved by higher yields, greater cropping intensities, and land expansion (SDSN, 2013). Since the access to mineral fertilizers in developing regions is very restricted, a sustainable intensification of land use can be addressed through the application of an integrated soil fertility management approach (ISFM), defined by Vanlauwe et al. (2010) as ‘a set of management practices that necessarily include the use of fertilizer, organic inputs and improved germplasm, combined with the knowledge on how to adapt these practices to local conditions, aiming at maximizing agronomic efficiency.’

The rhizosphere is populated by a diverse range of bacteria and fungi that positively influence plant growth. The implementation or stimulation of these indigenous plant growth-promoting (PGP) micro-organisms fits within the ISFM concept, but may require the development of proficient inocula via isolation and propagation of the most effective microbes (Sanginga and Woomer, 2009; Bender et al., 2016). In that context, major efforts have been made over the past decades to map the biodiversity and functionality of the organisms that collectively make up the rhizobiome (Kirk et al., 2004; Lakshmanan et al., 2014). For the fungi, ubiquitous endophytes appear to be arbuscular mycorrhizal fungi (AMF), dark septate endophytes (DSE), and Sebacinales (Blaalid et al., 2012; Detheridge et al., 2016), all exerting specific interactions with their hosts.

Arbuscular mycorrhizal fungi belonging to the phylum of the Glomeromycota are well-described because of their mutualistic symbiosis with the majority of land plants and their beneficial effects on plant growth under several adverse soil conditions, such as low nutrient status, drought, salinity, and high pathogen pressure (Smith and Read, 2008). The intraradical mycelium of AMF can branch into very thin hyphae to form the typical arbuscule structures inside root cortical cells, the main sites of nutrient exchange between plant and fungus. Intraradical hyphae can also produce vesicles, which are believed to function as storage organs. Positive effects on plant growth are mostly related to an improved nutrient and water uptake accomplished by the dense extraradical hyphal network that extends over long distances and allows the plant to access nutrients far beyond the normal nutrient depletion zone (Giovannetti et al., 2002; Parniske, 2008; Bonfante and Genre, 2010). However, AMF are obligate biotrophs that strictly depend on a living photoautotrophic partner for growth and production of spores (Bonfante and Genre, 2010), which makes the development of efficient inocula for use in the field rather difficult. In addition, not all plants are suitable AMF hosts. For instance, symbiotic associations are rarely established with members of the Brassicaceae, Chenopodiaceae, and Cyperaceae (Kuhad et al., 2004). Moreover, the success rate of inoculum application is highly dependent on soil conditions and colonization typically goes down with an increasing nutrient availability (Smith and Read, 2008).

The dark septate endophytes are a heterogeneous complex comprising a few orders of the phylum Ascomycota (Jumpponen and Trappe, 1998). DSE are characterized by the formation of melanized septate hyphae that grow inter- or intracellularly in the plant root and microsclerotia which are intracellular structures of irregularly lobed hyphae (Lukešová et al., 2015). DSE have a wide host range including non-mycorrhizal plants, and are found worldwide, especially in environments with strong abiotic stress (Jumpponen, 2001; Rodriguez et al., 2009). It is believed that DSE may also form mutualistic associations similar to those of AMF (Jumpponen, 2001), however, little is known about their actual role in plant ecosystems. Described effects on plant growth vary between positive or neutral (Newsham, 2011) and negative (Tellenbach et al., 2011). Unlike AMF, DSE are thought to lack specialized interfaces for nutrient transfer with the plant host. Hypotheses for the mode of action include mineralization of the substrate, production of phytohormones, and reduction of pathogen infestation (Lukešová et al., 2015).

Finally, within the order Sebacinales (Hymenomycetes) of the phylum Basidiomycota, *Piriformospora* (=*Serendipita*) *indica* that belongs to the recently proposed family Serendipitaceae (formerly Group B; Weiß et al., 2016), has attracted great interest since its discovery (Verma et al., 1998). *P. indica* is an endophyte with an exceptionally broad host range, including bryophytes, pteridophytes, gymnosperms, and a large number of angiosperms (Oelmüller et al., 2009; Qiang et al., 2012). Colonization in rhizodermal and cortical cells starts with a short biotrophic stage, followed by a cell death-associated phase and the production of intracellular pear-shaped chlamydospores (Jacobs et al., 2011). *P. indica* is able to colonize plant roots independent of soil phosphorus levels (Varma et al., 1999), and can be cultured on several synthetic media (Kost and Rexer, 2013). Its lack of host specificity, ease of inoculum propagation, and applicability under diverse conditions, combined with its stimulating effect on plant growth and yield and its capacity to confer systemic tolerance against (a)biotic stress, resulted in a strong interest for the implementation of *P. indica* as biofertilizer, bioprotector, and bioregulator, especially in environments characterized by extreme physical conditions and nutrient limitations (Varma et al., 2001, 2012; Waller et al., 2005; Oelmüller et al., 2009; Franken, 2012). Different environmental molecular analyses have shown the ubiquitous presence of Sebacinales and *Piriformospora* within the roots of terrestrial plants worldwide (Selosse et al., 2009; Weiß et al., 2011). Nevertheless, only a limited number of sebacinalean fungi has been successfully cultured (Weiß et al., 2016), possibly suggesting difficulties with direct isolation from field samples. Recently, *P. williamsii* was described as the second *Piriformospora* representative (Basiewicz et al., 2012). In contrast to *P. indica*, the PGP activity of this species has been reported as absent or limited on *Arabidopsis* (Lahrmann et al., 2013; Banhara et al., 2015) but strong on barley (Sharma and Kogel, 2009). Together with *S.* ‘*vermifera*’ (species complex) and *S. herbamans*, *P. indica*, and *P. williamsii* are the only four currently accepted species within the newly defined Serendipitaceae (Weiß et al., 2016).

To improve insights into their mode of action and extend their potential applications in agriculture and biotechnology, it would be highly beneficial if more isolates of the Sebacinales would be available. Therefore, in this study, efforts were made to isolate novel PGP fungi from trapping systems with rhizospheric soils from fields of Kisangani in the Democratic Republic of Congo (DRC) as starting inoculum and with sudangrass as a host. The resulting collection of 51 axenic *Piriformospora* cultures was phenotypically and molecularly analyzed and represents a valuable contribution to the Serendipitaceae.

## Materials and Methods

### Origin and Sampling of Rhizospheric Soil

**FIGURE 1:**
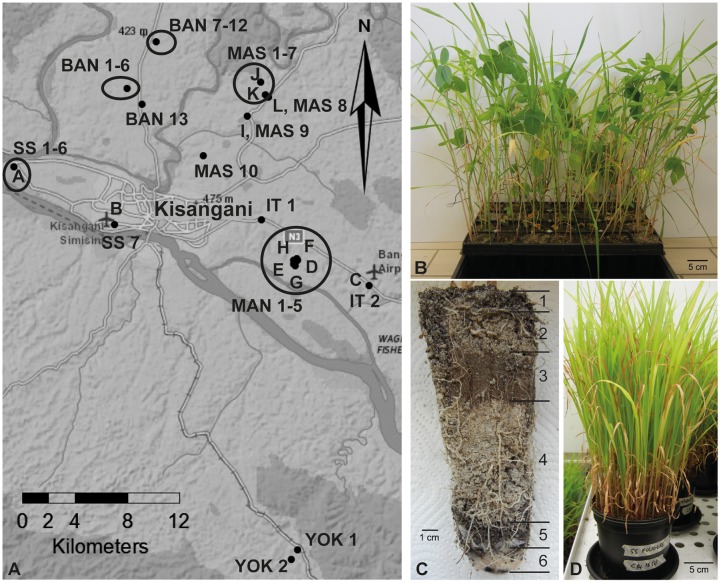
**Sampling sites and experimental setup of soil-based trap systems. (A)** Map of the region Kisangani in the Democratic Republic of Congo where rhizospheric soil samples were collected. Samples A–L were collected during the first sampling round, and samples BAN, IT, MAN, MAS, SS, and YOK during the second round. BAN, route Banalia; IT, route Ituri; MAN, Mandombe; MAS, route Masako; SS, route Simi-Simi; YOK, Yoko. **(B)** Tray trap system with four different crops (maize, rice, soybean, and sudangrass). **(C)** Layering in a tray trap system; from top to bottom: lava beads (1), a 3:2 Terragreen: sand mixture (2), inoculum soil layer (3), a 3:2 Terragreen: sand mixture (4), lava beads (5), cotton plug (6). **(D)** Sudangrass-based trap system in bigger pots.

A first sampling was performed in August 2013 in the rural areas surrounding the city of Kisangani (0.51528 N, 25.19099 E, and 447 m above sea level), which is located in the Northeast of the DRC. Twelve soil samples were collected from the rhizosphere of various plants at six different sites (samples A–L; **Figure 1A**; Supplementary Table S1).

Additional sampling rounds were organized in March and August 2014 along the main axes exiting the city of Kisangani (samples BAN, IT, MAN, MAS, SS, YOK; **Figure 1A**). More sampling sites and crops were included, with 39 samples as a result (Supplementary Table S1). Each time, entire root systems were excavated and adhering soil with fine roots was collected.

The rhizospheric soil samples were preserved at 4°C and transported to Belgium (Faculty of Bioscience Engineering, Ghent University) to serve as substrate in greenhouse trap experiments.

### Host Plants and Seed Germination

Different crops served as hosts in the trap systems to facilitate colonization by as many endophytic fungi as possible. Maize (variety Kasai), rice (variety Nerica) and soybean (variety Viscoza 000) seeds were purchased locally in Kisangani; sudangrass [*Sorghum sudanense* (Piper) Staph., Karim] was provided by Neutkens seed company (Vessem, The Netherlands). Seeds were surface sterilized with 70% ethanol for 2 min followed by 2.5% sodium hypochlorite (NaOCl) under continuous shaking for 5 or 15 min for maize/soybean and rice/sudangrass, respectively. Sterilized seeds were rinsed five times with sterile distilled water and pre-germinated on Whatman no 1 filter paper at 27°C in the dark for 3 days.

*Arabidopsis thaliana* (accession Col-0) seeds for *in vitro* experiments were surface sterilized by incubation for 3 min in 70% ethanol with 0.05% Triton X-100, followed by 10 min in 100% ethanol and drying.

### Experimental Setup of Different Trapping Systems

The first trapping system was a tray system with the rhizospheric soil samples of the first sampling round as inoculum and with maize, rice, soybean, and sudangrass as host plants to facilitate colonization by different endophytic fungi. Modiform trays type 2520 (Intergrow, Aalter, Belgium) with a volume of 370 ml and a depth of 15 cm (**Figure 1B**) were filled with different layers of substrate in the following sequence from bottom to top: sterile cotton plug, 1-cm layer of sterile 0-to-3-mm lava beads (DCM, Grobbendonk, Belgium), sterile 3:2 mixture of Terragreen (oil dry US special, type III R, <0.125 mm, Lobbe Umwelttechnik, Iserlohn, Germany) and quartz sand to fill the tray halfway, 85 g of wet rhizospheric soil sample, 2-cm layer of Terragreen:sand mixture (3:2) and 1-cm layer of lava beads (**Figure 1C**). For the non-inoculated treatments, trays without soil or with autoclaved soil were used. Three seedlings were planted per tray and one repetition was installed per soil sample and per crop. Plants were maintained in a greenhouse under a temperature regime of 28/23°C (day/night) and a relative air humidity of 80% (irradiated 12 h per day) for 6 weeks.

To obtain pure *Piriformospora* isolates, other trap systems were set up with rhizospheric soils from the second sampling round, using only sudangrass as superior host. The same substrate succession and growth conditions were used as described above. The choice of bigger pots with a volume of 2 L (**Figure 1D**) allowed that plants could be grown for a longer period. Every 10 months, new sudangrass secondary trap systems were initiated, using substrate and chopped roots of the previous trap as inoculum source for the new one. In that way, a plant-based collection was maintained to anticipate possible activity losses of the *Piriformospora* isolates. Pots were watered twice a week with distilled water and fertilized with a low-phosphorus Hoagland solution (Hoagland and Arnon, 1950) every 6 weeks. Scoring of colonization was done after 6–8 months, at harvest time (after 10 months) and again 6 weeks after installation of secondary trap cultures.

### Visualization of Root-Associated Fungi

Plant roots were harvested and stained according to a modified protocol of the ink-vinegar technique of Vierheilig et al. (1998). Roots were first rinsed under running tap water to remove adhering soil particles. Next, roots were depigmented with a 10% KOH solution. After incubation at 80°C for 30 min, the solution was poured off and replaced by a 1% HCl solution with a rinsing step in between. Roots were acidified for 20 min at room temperature before adding droplets of Parker Quink blue ink (2 droplets per 25 ml). This mixture was incubated at 80°C for 5 min and at room temperature for an additional 30 min. Finally, the staining solution was removed, roots were rinsed with water and an acidified glycerol solution (700 ml glycerol + 230 ml water + 70 ml 1% HCl) was added to extract the ink stain from the plant cells. Stained roots were observed microscopically with a Motic SMZ-168 stereo microscope (Motic, Causeway Bay, Hong Kong) and an Olympus BX41 light microscope (Olympus America, Inc., Center Valley, PA, USA) for presence of endophytic fungi.

### Isolation and Growth Conditions of Endophytic *Piriformospora* from Sudangrass Trap Roots

Roots from sudangrass trap systems were used to isolate *Piriformospora* endophytes at different time points (6 and 8 months after initiation of the first trap; 6 weeks after initiation of the secondary trap). Therefore, chlamydospore-containing root samples were washed in a stream of running tap water to remove adhering substrate particles. Next, the sample was soaked in 70% ethanol for 1 min, followed by immersion in 2.5% NaOCl for 3 min. The surface sterilized roots were rinsed at least five times with sterile distilled water and cut in 1-cm pieces. These root fragments were incubated in the dark at 22°C on six different media supplemented with 300 μg/ml of the antibiotic spectinomycin: complex medium (CM), malt extract agar (MEA), malt yeast peptone (MYP), Modified Melin-Norkrans (MMN), potato dextrose agar (PDA) and 1.5% water agar (Supplementary Table S2). The plates were monitored every day for fungal growth and a selection was made for colonies with colorless non-aerial hyphae growing on the surface of the medium. Candidate *Piriformospora* isolates were maintained on PDA medium at 27°C until further processing.

### Preparation of Spore Suspensions and Long Term Storage of *Piriformospora* Isolates

Chlamydospores were harvested from 4-week-old PDA culture plates by flooding the surface with a 0.02% (vol/vol) aqueous solution of Tween 20 followed by scraping the plate with an inoculation loop. This procedure was repeated twice to obtain a total spore suspension of approximately 15 ml which was filtered through a sterile cotton plug to remove particles of mycelium. The spore filtrate was centrifuged at 8,500 rpm for 15 min and the pellet resuspended in phosphate buffered saline (PBS). Spore concentrations were determined with a Bürker counting chamber and the suspensions were diluted to a final concentration of 5 × 10^5^ spores/ml.

Monospore cultures were produced for storage of pure isolates. The cotton-wool-filtered spore suspension was further diluted to a concentration of 5,000 spores/ml of which 100 μl was plated on CM medium. This was incubated at 30°C for 2–3 days until spore germination had started. Single germinating spores were selected under an Olympus BX41 light microscope, excised from the medium and transferred to a new PDA Petri dish. The resulting monospore cultures were incubated at 30°C for 4 weeks. Equal amounts (vol/vol) of 20% glycerol and undiluted spore suspensions from monospore plates, obtained as described above, were mixed in 2-ml low temperature freezer vials and stored at -80°C.

### Morphological Characterization of the Isolates

Spore stocks preserved at -80°C were used to initiate fresh cultures of the *Piriformospora* isolates. The colony morphology was assessed in triplicate on four different media (CM, MEA, MYP, and PDA) at 30°C. Special attention was given to growth patterns of the fungal colonies, and color and structure of the hyphae. At days 2, 5, 7, 9, and 12 after inoculation of the plates, the colony surface area (cm^2^) was determined as a parameter for the growth rate.

In addition, the average size (length) of the spores produced by 4-week-old cultures grown on PDA was measured from two biological replicates set up independently in time (*n* = 50). Spore images were taken on an Olympus IX81 microscope equipped with an Olympus XC50 digital camera, using the Cell^∗^ Imaging software, and were imported in the image processing tool ImageJ to do calibrated measurements (Schneider et al., 2012).

### Molecular Identification of Sebacinales

DNA was extracted from colonized roots (approximately 100 mg) or from freeze-dried mycelium with the Invisorb Spin Plant Mini Kit according to the manufacturer’s instructions (Isogen, De Meern, The Netherlands). For the latter, isolates were cultured in 24-well plates, each well containing 1 ml potato dextrose broth (PDB). After 6 days of growth at 27°C, the mycelium was harvested and subjected to vacuum freeze-drying.

The Sebacinales identity of the isolates was verified by the specific PCR amplification of a ∼2,200–2,500 bp DNA fragment comprising the 3′ region of the small subunit (SSU; 18S), the internal transcribed spacer 1 (ITS1), the 5.8S subunit, ITS2, and the D1/D2 region of the large subunit (LSU; 28S) of the ribosomal DNA with the primers NSSeb1 and NLSeb2R. For sequencing purposes, this PCR was followed by a second PCR with the universal fungal primers ITS1F and NL4 (Supplementary Table S3). The 20 μl PCR mix of both reactions included 20 ng genomic DNA or 1 μl from a 1:100 dilution of the first amplification products respectively, 1x Phusion GC buffer (Thermo Fisher Scientific, Erembodegem, Belgium), 0.2 mM dNTP mix (Promega, Leiden, The Netherlands), 0.25 μM of each primer (IDT, Leuven, Belgium) and 0.4 U Phusion High-Fidelity DNA polymerase (Thermo Fisher Scientific). PCR conditions were as described in Garnica et al. (2013), with the exception of the denaturation temperature that was augmented to 98°C (for 15 s in each cycle). The ∼1,250 bp amplicons of the nested PCR were purified using the E.Z.N.A. Cycle Pure Kit according to the manufacturer’s instructions (Omega Bio-tek, Norcross, GA, USA) and sequenced by LGC Genomics (Berlin, Germany) with the primers ITS1F, NL4, NL1, NLB4, ITS2, and ITS3 (Supplementary Table S3).

For a second DNA marker analysis, a ∼1,150 bp fragment of the translation elongation factor 1-α (TEF1α) gene was amplified with primers EF1-983F and EF1-2218R (Supplementary Table S3). The 25 μl PCR reaction included 20 ng genomic DNA, 1x Green GoTaq reaction buffer (Promega), 0.2 mM dNTP mix (Promega), 0.2 μM of each primer (IDT) and 0.625 U GoTaq polymerase (Promega). A touchdown PCR procedure was done according to Rehner and Buckley (2005). For sequencing, two additional internal primers, 1567R and 1577F, were used (Supplementary Table S3).

Consensus sequences were deposited in GenBank under the accession numbers KY509316–KY509330.

### ISSR Analysis of the Isolates

An initial set of 15 inter-simple sequence repeat (ISSR) primers, often used in fungal diversity studies (Supplementary Table S4), was tested for its applicability in generating profiles with a sufficient number of countable bands (>8) of which at least 70% were polymorphic. *P. williamsii* and four Congolese isolates were used for this screening. The 25 μl PCR reaction included 10 ng genomic DNA, 1x Green GoTaq reaction buffer (Promega), 0.2 mM dNTP mix (Promega), 0.5 μM primer (IDT) and 0.625 U GoTaq polymerase (Promega). The thermocycling program consisted of initial heating at 94°C for 5 min, followed by 40 cycles of denaturation at 94°C for 1 min, annealing at 52°C for 1 min and extension at 72°C for 2 min, followed by a final extension at 72°C for 8 min. The 1.5% gel was run at 100 V for 2.5 h. After this first screening, a subset of four primers was retained for further analysis.

Then, a gradient PCR with temperatures ranging from 46°C up to 56°C was done to determine the optimal annealing temperature for each of the four primers: 48°C for (AC)_8_YG, 50°C for (GA)_8_YG and (AC)_8_C, and 52°C for (GA)_8_YC.

Subsequently, all 51 Congolese isolates, and *P. indica* and *P. williamsii* as references, were analyzed with the selected primers under above described conditions. This was repeated for a subset of nine isolates, including the *Piriformospora* references. MassRuler DNA Ladder Mix (Thermo Fisher Scientific) was loaded every 5–6 samples to allow the digital alignment of the banding profiles using the Applied Maths software BioNumerics (version 5.1; Applied Maths, Sint-Martens-Latem, Belgium). A dendrogram was constructed for all four primers separately using the unweighted-pair group method with arithmetic mean (UPGMA) which is based on the Pearson correlation similarity coefficient (Sokal and Rohlf, 1981).

Considering the identical banding profiles resulting from the 2 or 3 (including initial screening) technical repetitions to which the selection of isolates was subjected, reproducibility across time could be demonstrated. In addition, with two different DNA extractions of *P. indica*, the reproducibility of biological repetitions was also verified.

### Plant Growth Promotion Assay

Sterilized *Arabidopsis thaliana* Col-0 seeds (see paragraph on host plants and seed germination) were transferred to a square Petri dish (12 cm × 12 cm) containing 35 ml of half-strength Murashige and Skoog (MS) medium without sucrose (Supplementary Table S2). After 4 days at 4°C, the plates with vernalized seeds were placed vertically in a growth chamber (22°C, 16 h light/8 h dark) for 5 days. By means of a Drigalski spatula, 50 μl aliquots of the chlamydospore suspensions (30,000 spores) of the different isolates were spread over the entire width of the Petri dishes at a distance of 3.5 cm below the seedlings. Controls were mock-treated with 50 μl of PBS solution. The experiment was repeated twice with two and three technical replicates, respectively.

Nine days after inoculation, the shoot fresh weight was determined for each individual plant and the leaf surface area was measured for the entire plate using ASSESS 2.0 Image Analysis Software (Lamari, 2002). To facilitate the quantification of the total root length (main plus lateral roots) and the number of lateral roots per plate, a dedicated software tool was written in Python (Supplementary Materials and Methods; Supplementary Figure S1).

### Statistical Analysis

Statistical analyses were done in SPSS statistics, version 23 (SPSS, Inc., Chicago, IL, USA). For each experiment the data were first checked for normality of the response variable per isolate (Shapiro–Wilk test) and homoscedasticity of variances. For colony surface area and spore length, significant deviations from the normality assumption were observed. Therefore, a Kruskal–Wallis test was carried out to determine overall differences, followed by a Dunn’s test adjusted for multiple comparisons. The data of *Arabidopsis* shoot fresh weight, leaf surface area, total root length and number of lateral roots met the assumptions of normality and homoscedasticity, and were hence analyzed using one-way analysis of variance (ANOVA), followed by a one-sided Dunnett’s test to compare each treatment with the control. For each test, statistical significance was set at a level of α = 0.05.

## Results and Discussion

### Sudangrass-Based Systems Trap *Piriformospora* from Rhizospheric Soil Samples

**FIGURE 2:**
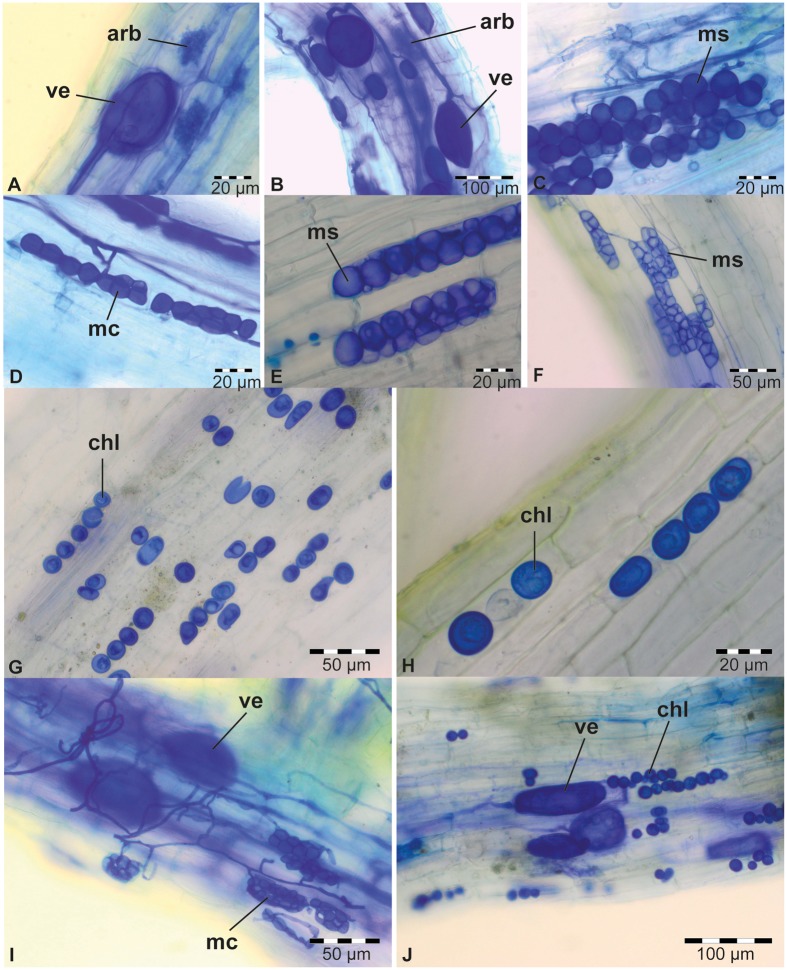
**Typical colonization of plant roots from 6-week-old trap experiments**. AMF in sudangrass of trap culture E **(A)** and in maize of trap B **(B)**. DSE in sudangrass of trap E **(C)**, rice of trap L **(D)**, maize of trap F **(E)**, and sudangrass of trap F **(F)**. *Piriformospora* chlamydospores in maize **(G)** and sudangrass **(H)** of trap culture D. **(I)** Co-occurrence of DSE and AMF in sudangrass of trap E. **(J)** Co-occurrence of *Piriformospora* and AMF in maize of trap D. All plant roots were stained with Parker blue ink. Arb, arbuscules (AMF); chl, chlamydospores (*Piriformospora*); mc, moniliform cells (DSE); ms, microsclerotia (DSE); ve, vesicles (AMF).

To screen the sampled rhizospheric soils for the presence of PGP fungi, a soil-based trap system was used in combination with maize, rice, soybean, and sudangrass as hosts (**Figure 1B**), four crops that are abundantly cultivated in sub-Saharan Africa. As a test for the experimental set-up, 12 soil samples collected from different plants at six different sites around Kisangani (**Figure 1A**; samples A–L; Supplementary Table S1) were included as natural inoculum in the traps (**Figure 1C**). Six weeks later, the roots of the four crops were microscopically scored for the presence of visible structures of endophytic fungi (**Figure 2**).

For soybean and rice, respectively, only 17 and 25% of the soil samples resulted in root colonization by endophytic fungi in our tray system (**Table 1**). In contrast, maize (58%) and especially sudangrass (83%) roots were much more efficiently colonized by AMF and DSE, as shown by the presence of arbuscules and/or vesicles (**Figures 2A,B**), and microsclerotia and/or moniliform cells (**Figures 2C–F**), respectively (**Table 1**). The poor performance of rice compared to maize and sudangrass in our experiments, could possibly be explained by the limited development of the rice roots at harvest time. Indeed, one of the requirements for successful endophyte establishment is a well-developed plant root system. Moreover, whereas maize and sudangrass have a high mycorrhizal potential, soybean is rarely mentioned as a host in soil-based trap systems as opposed to other Fabaceae such as *Trifolium* species, suggesting that it is a poor host (Brundrett et al., 1996; Stutz and Morton, 1996; Ezawa et al., 2000; Liu and Wang, 2003; Ijdo et al., 2011).

**Table 1:** Scoring of endophyte establishment in trap experiments with four different host crops and 12 different soil samples from the Kisangani area as inoculum.

Sample code	Maize			Rice			Soybean			Sudangrass		
	AMF	DSE	PIRI	AMF	DSE	PIRI	AMF	DSE	PIRI	AMF	DSE	PIRI
A	-	-	-	-	-	-	-	-	-	-	-	-
B	+	-	-	-	-	-	-	-	-	+	+	-
C	-	-	-	-	-	-	-	-	-	-	+	-
D	+	-	+	-	-	-	-	-	-	+	-	+
E	+	+	-	-	-	-	-	-	-	+	+	-
F	-	+	-	-	-	-	-	-	-	-	+	-
G	-	-	-	-	-	-	-	-	-	-	-	-
H	+	-	-	+	-	-	+	-	-	+	-	-
I	-	-	-	-	-	-	-	-	-	+	-	-
J	-	-	-	-	-	-	-	-	-	-	+	-
K	-	+	-	-	+	-	-	-	-	-	+	-
L	+	+	-	-	+	-	-	+	-	+	+	-

*After 6 weeks, the roots of maize, rice, soybean, and sudangrass were stained with Parker blue ink and scored for the presence (+) or absence (-) of arbuscular mycorrhizal fungi (AMF), dark septate endophytes (DSE), and *Piriformospora* (PIRI)*.

Interestingly, the presence of one endophyte apparently did not prevent another one from colonizing the same root piece, since in several roots AMF and DSE coexisted (**Figure 2I**; **Table 1**). Co-occurrence of AMF and DSE has been reported before (Lingfei et al., 2005; Mandyam and Jumpponen, 2005; Postma et al., 2007; Wagg et al., 2008; García et al., 2012), but its role in ecosystems is still poorly understood.

Surprisingly, with soil sample D, maize and sudangrass roots contained structures resembling chlamydospores, indicative of *Piriformospora* (**Figures 2G,H**; **Table 1**), and co-occurrence of these structures with AMF was also observed (**Figure 2J**). Interestingly, the single available isolates of *P. indica* and *P. williamsii* were reported to be obtained from AMF spores (Williams, 1985; Verma et al., 1998), suggesting that both fungi can live in close association. Genomic DNA was prepared from chlamydospore-containing roots and the identity of the putative *Piriformospora* fungi was verified through a Sebacinales-specific amplification of a part of the ribosomal DNA (Garnica et al., 2013). An amplicon of the expected size was obtained (Supplementary Figure S2A) and sequencing revealed that the Congolese endophytes were indeed *Piriformospora*. Multiple sequence alignments of the obtained sequences indicated that the endophytes were closely related to, but different from *P. williamsii* and *P. indica* (Supplementary Figure S3). Although several molecular studies have shown the widespread distribution of sebacinoid fungi in several plant species worldwide (Selosse et al., 2009; Weiß et al., 2011), the majority of the more than 7,000 Sebacinales DNA and RNA sequences deposited in GenBank are from environmental samples, indicating that isolating these fungi is problematic. Indeed, to date, only single isolates of the two *Piriformospora* species are available to the scientific community, making our finding particularly exciting.

### Acquisition of 51 *Piriformospora* Isolates from Congolese Rhizospheric Soils

Next, 39 fresh rhizospheric soil samples collected in the area of Kisangani (BAN 1-13, IT 1-2, MAN 1-5, MAS 1-10, SS 1-7, YOK 1-2) were used to initiate trap systems with larger pots and sudangrass as a superior host (**Figures 1A,D**; Supplementary Table S1). The trap roots were screened at different time points for the presence of *Piriformospora*. Based on the occurrence of either chlamydospores or vesicles inside the roots, 18 and 16 soils were scored positive for the presence of *Piriformospora* or AMF, respectively (Supplementary Table S1). In 11 samples, both endophytes had colonized the roots simultaneously, confirming our initial observation on the coexistence of *Piriformospora* and AMF. Although Mandombe appeared to be the most conducive location with *Piriformospora* colonization in each of the five traps, chlamydospores could also be detected in at least one of the traps for the other locations of the sampled area (15-km radius). Moreover, the sampled soils represented rhizospheres of very diverse plants, including banana, *Chromolaena odorata*, fern, maize, pineapple, Poaceae sp., *Pueraria javanica* and sugarcane (Supplementary Table S1), demonstrating the lack of a strong host preference as described before for *P. indica* and *P. williamsii* (Varma et al., 2001; Oelmüller et al., 2009; Basiewicz et al., 2012). Altogether, these findings suggest that *Piriformospora* is an ubiquitous fungus in the Kisangani area.

Sudangrass roots of the most strongly colonized traps were subjected to an isolation procedure. *Piriformospora* mycelium could be obtained from the roots of six traps (Supplementary Figure S2B), resulting in a total collection of 51 isolates (Supplementary Table S1): seven from MAS 2 (isolates 1–7), 16 from SS 3 (isolates 8–23), two from MAN 2 (isolates 24–25), five from MAN 3 (isolates 26–30), eight from MAS 6 (isolates 31–38), and 13 MAS 9 (isolates 39–51).

### Effect of (Long Term) Storage on the Viability of the Isolates

Initially, Petri dishes with 4-week-old PDA cultures were transferred to 4°C for preservation of the mycelium and spores. However, after 2 months of storage, it was observed that viability of the isolates was reduced or completely lost. For that reason, cultures on solid medium were kept at room temperature (22°C), which did not affect growth capacity as evidenced by a successful subculturing of the isolates after 3 months.

Furthermore, the colonization efficiency of *Piriformospora*-positive rhizospheric soil inoculum was seriously impaired by cold storage at 4°C for several weeks (data not shown).

In contrast, preservation of chlamydospores in 10% glycerol at -80°C seems to be a suitable conservation method. Preserved spores were plated on PDA after 3, 6, 9, and 12 months of storage, and germination capacity was not affected (data not shown). Possibly, improper storage of samples prior to analysis might partially explain why isolation of these fungi has proven to be difficult, although it has to be noted that *P. indica* seems to survive UK weather conditions well (Rabiey et al., 2017).

### The Congolese *Piriformospora* Isolates Are Morphologically Diverse

**FIGURE 3:**
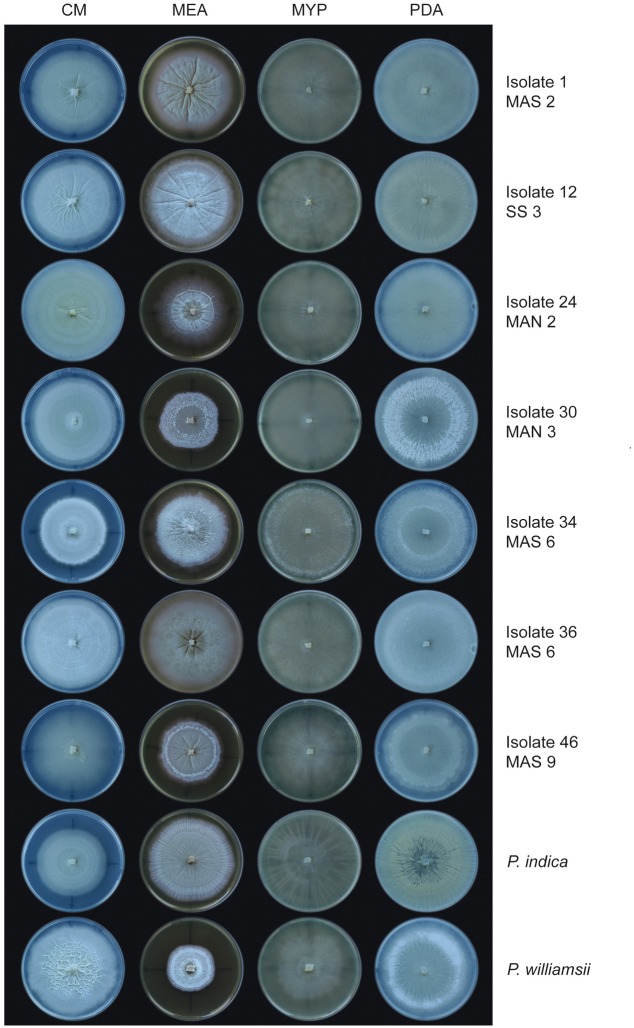
**Colony morphology of *Piriformospora* isolates on different media**. Seven Congolese isolates (1, 12, 24, 30, 34, 36, and 46), and *P. indica* and *P. williamsii*, were grown on four different media for 12 days at 30°C. Plates were sealed with Parafilm. On MEA, isolate 1 and all other cultures from sampling site MAS 2 displayed a smooth and creamy-colored wrinkled colony with strong radial furrows. On CM, isolate 46 and all other cultures from MAS 9 were typically characterized by an irregular star-like polygon in the center of the plate. CM, complete medium; MEA, malt extract agar; MYP, malt yeast peptone agar; PDA, potato dextrose agar.

The biodiversity of the 51 isolates was first assessed by evaluating the macroscopic morphology of their mycelium on different media (CM, MEA, MYP, and PDA) and comparing it to that of *P. indica* and *P. williamsii*. Although clear differences were observed between isolates and media (**Figure 3**), isolates from individual trap cultures had very similar appearances on particular media, suggesting that the plants were colonized by single morphotypes. The only exception was the trap with soil sample MAS 6, in which two different types of isolates could be detected (**Figure 3**). Importantly, none of the isolates exhibited the same macroscopic growth patterns on the different media as the reference strains. Irrespective of the medium, however, their mycelium was usually submerged in the agar with limited formation of aerial hyphae. Moreover, in liquid medium the mycelium was conglomerated in small globose balls (Supplementary Figure S4). These two characteristics are typical for *Piriformospora* species (Verma et al., 1998; Basiewicz et al., 2012; Kost and Rexer, 2013). Based on their morphological features, seven isolates representing all sampling locations, were randomly chosen for further analysis (**Figure 3**).

**FIGURE 4:**
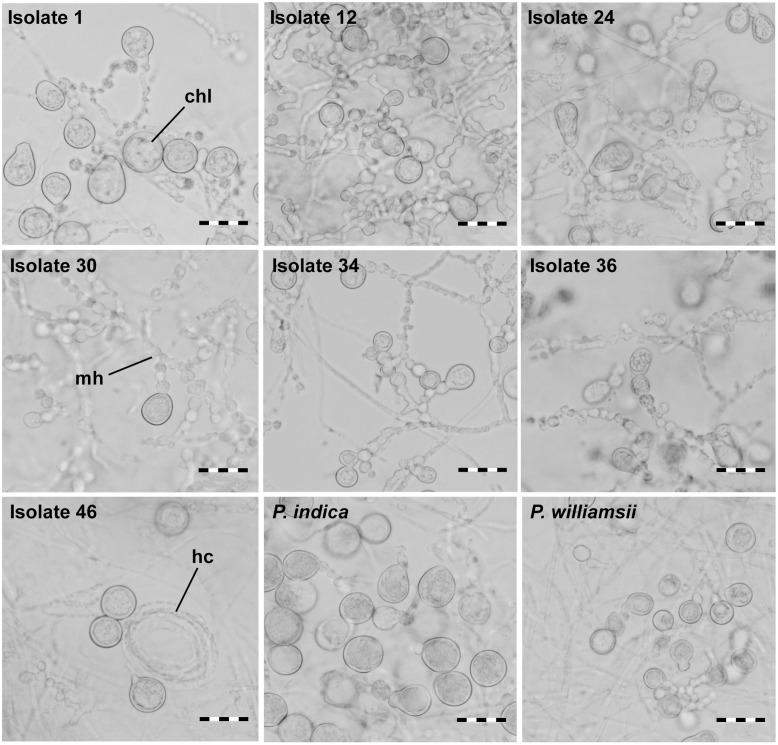
**Spore morphology of *Piriformospora* isolates using bright field microscopy**. Isolates were grown for 4 weeks on solid PDA medium; spore formation at the tips of moniloid hyphae was observed. Chl, chlamydospore; hc, hyphal coil; mh, moniloid hyphae. scale bar = 20 μm.

**FIGURE 5:**
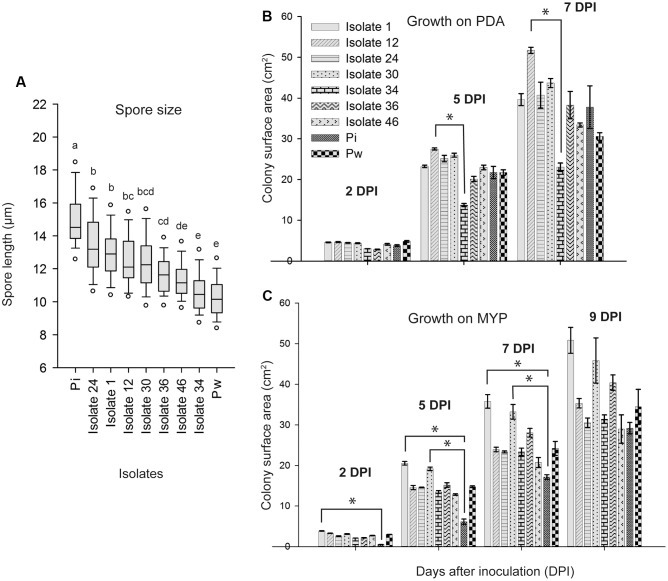
**Spore length and growth rate of *Piriformospora* isolates. (A)** Box-plot of the spore length of seven Congolese isolates (1, 12, 24, 30, 34, 36, and 46), and *P. indica* (Pi) and *P. williamsii* (Pw). Spores were harvested from 4-week-old PDA culture plates grown at 30°C. Isolates indicated with the same letter have a similar spore size range according to Dunn’s test adjusted for multiple comparisons (α = 0.05). **(B,C)** Colony surface area (cm^2^) measured 2, 5, 7, 9 days post inoculation (dpi) of PDA **(B)** and MYP **(C)** medium. Error bars indicate standard errors on the mean of three replicates. Asterisks point out statistical significant differences between treatments (Dunn’s test adjusted for multiple comparisons α = 0.05). Overall, the growth was faster on PDA than on MYP and there was a strong variation among the isolates and the reference strains. For instance, whereas on PDA isolate 12 grew fastest and isolate 34 slowest, on MYP the fastest were isolates 1 and 30 and the slowest *P. indica*. MYP, malt yeast peptone agar; PDA, potato dextrose agar.

When observed microscopically, intertwined, young mycelium was a white to hyaline tubular structure without indentations. However, as the mycelium aged, it had a granular appearance and was more irregularly inflated (moniliform). Hyphae were irregularly septated and sometimes arranged in multilayered coils. Single or clustered chlamydospores sprouted at the tips of the hyphae and had a globular to pear-shaped form (**Figure 4**). No clamp connections or sexual structures (basidia) were observed. The spore length of the seven Congolese isolates ranged between that of *P. williamsii* that had the smallest spores (9–12 μm) and that of *P. indica* that had the largest spores (13–18 μm) (**Figure 5A**). Based on spore width, the same ranking of the isolates was obtained as that based on spore length (data not shown).

Finally, growth rate was assessed on MYP and PDA because on these media the isolates displayed the most uniform colony morphology (**Figure 3**). Overall, the growth was faster on PDA than on MYP and there was a strong variation among the isolates and the reference strains (**Figures 5B,C**).

Altogether, the phenotypic features of the selected isolates suggest that they might be genetically different from each other and from the reference strains. Nonetheless, they exhibit typical characteristics of *Piriformospora* and appear to be more similar to *P. williamsii* than to *P. indica*. We serendipitously discovered that *P. williamsii* can be distinguished from *P. indica* because it scores positive for catalase activity whereas *P. indica* does not. Therefore, a drop of 3% H_2_O_2_ was placed on the mycelium of PDA-grown fungi and catalase activity was assessed by the formation of gas bubbles. *P. williamsii* as well as the seven selected Congolese isolates immediately formed bubbles, while for *P. indica* no response was observed, supporting a closer relationship of the isolates to *P. williamsii*. According to Basiewicz et al. (2012), a differential activity between the two reference strains is also detected for peroxidase, but in that case *P. indica* is positive and *P. williamsii* is negative.

### ISSR Analysis Confirms the Biodiversity of the Congolese Isolates

For genetic evidence on the biodiversity of the 51 isolates, ITS and TEF1α amplification and sequencing was done. Although single amplicons of the expected size were obtained, for both barcode genes the sequencing chromatograms sometimes displayed a series of ‘double peaks’ of approximately equal intensity starting at a particular point in the sequence (Supplementary Figure S3). In addition, single nucleotide polymorphisms (SNPs) could be observed as ambiguous sites with two different nucleotides at the same position at a few isolated locations in the sequences. Analysis of the superimposed sequences (‘double peaks’) with the Mutation Surveyor software (Minton et al., 2011) indicated the occurrence of length variant polymorphisms or heterozygous indels, implying the presence of two different alleles varying by an insertion or deletion of several nucleotides. Indeed, based on genome size information combined with SNP analysis, Zuccaro et al. (2011) concluded that *P. indica* is most likely a heterokaryon containing two genetically distinct nuclei. Although cloning can resolve the different alleles, addressing this issue is beyond the scope of this study. Nevertheless, multiple sequence alignments of trimmed *ITS* and *TEF1α* amplicons (without superimposed regions) were generated which confirmed the position of the Congolese isolates within the *Piriformospora* cluster of the Serendipitaceae (Supplementary Figure S3). The alignments also suggested that the new isolates and *P. indica* belong to different species, because their genetic distance was comparable to that of the two *Piriformospora* reference strains. Nevertheless, is has to be noted that the separation of *P. williamsii* from *P. indica* is based on very sparse sampling of conspecifics (*n* = 1) within the clade. The resolving power of our data was too low to detect sufficient genetic diversity among the new isolates and between the latter and *P. williamsii*.

Therefore, as an alternative approach to assess biodiversity, inter-simple sequence repeat (ISSR) analysis was chosen as a fast, reliable, and genome-wide technique (Sarwat, 2012). A set of 15 primers was first tested on a selection of five isolates for its applicability in generating profiles with a sufficient number of polymorphic bands (Supplementary Table S4). The number of countable amplified bands ranged from 6 to 17 and the percentage of polymorphism from 40 to 89%. From this first screening, a subset of four primers displaying at least 70% polymorphism was retained for analysis of the 51 isolates. Inclusion of more isolates in the ISSR resulted in a higher number of polymorphic bands. The final percentages of polymorphism were 95% for (AC)_8_YG, 100% for (GA)_8_YG, 100% for (AC)_8_C, and 100% for (GA)_8_YC.

The ISSR fingerprints of the 51 isolates obtained with the four primers showed that isolates originating from the same rhizospheric sample were (nearly) identical. In contrast, polymorphisms could be detected among isolates recovered from the six different traps (Supplementary Figures S5A–D). Moreover, for the isolates of trap culture MAS 6, two banding profiles were generated with the primers (AC)_8_YG and (GA)_8_YC. Thus, in full agreement with the morphological data, seven different ‘genetic groups’ can be distinguished amongst the 51 isolates. This high biodiversity is remarkable since the sampling radius was maximum 15 km (when Yoko is not taken into account) and some sites, such as MAN 2 and MAN 3, were only a couple of meters apart. These findings indicate that, although different crops apparently harbor specific morphotypes, multiple isolates can be present in a single area, implying that Congolese soils might be a rich source of novel *Piriformospora* species.

**FIGURE 6:**
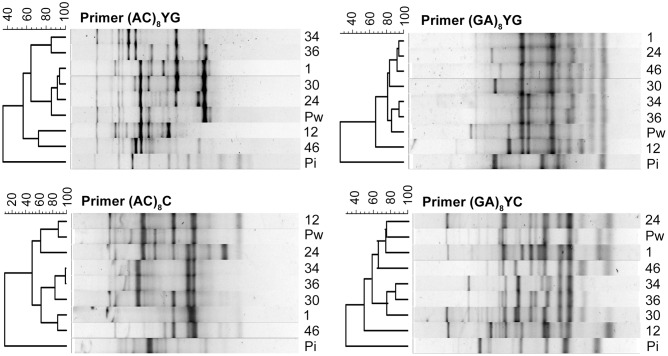
**Relatedness among *Piriformospora* isolates revealed by UPGMA analysis of ISSR profiles**. ISSR-based dendrograms were constructed by BioNumerics software for the primers (AC)_8_YG, (GA)_8_YG, (AC)_8_C, and (GA)_8_YC. Values above the ISSR profiles show percentage similarity inferred by Pearson correlation coefficient. Pi, *P. indica*; Pw, *P. williamsii*; 1-12-24-30-34-36-46, Congolese isolates.

The seven isolates that were selected for the morphological characterization are representatives of each of the above mentioned ‘genetic groups,’ and were used to repeat the ISSR procedure for phylogenetic analysis (Supplementary Figure S5E). The resulting banding profiles were used to construct UPGMA dendrograms with BioNumerics software. For each of the four primers, the percentage similarity inferred by the Pearson correlation coefficient between the Congolese cluster and *P. indica* was very low, with values ranging from 9.1% for primer (AC)_8_C to 35.3% for primer (AC)_8_YG, supporting that they might represent different species (**Figure 6**). In contrast, *P. williamsii* always clustered together with the Congolese isolates, and within this cluster the percentage similarity varied between 49.3% (primer (GA)_8_YC) and 98.5% (primer (AC)_8_C) (**Figure 6**). However, since different isolates of the same fungal species can generate profiles with polymorphic bands (Wang et al., 2005), no conclusive statements on their taxonomic position can be made. Nevertheless, the observed morphological variation on different media, together with their strong molecular similarity, argue in favor of the existence of a species complex, a selected group of closely related almost indistinguishable strains with an uncertain taxonomic status (Chen et al., 2016). In analogy with the species complex *Serendipita* ‘*vermifera*’ (Weiß et al., 2016), we propose to designate the Congolese collection as *Piriformospora* ‘*williamsii.*’

### The Congolese *Piriformospora* Isolates Strongly Promote *Arabidopsis* Seedling Growth

**FIGURE 7:**
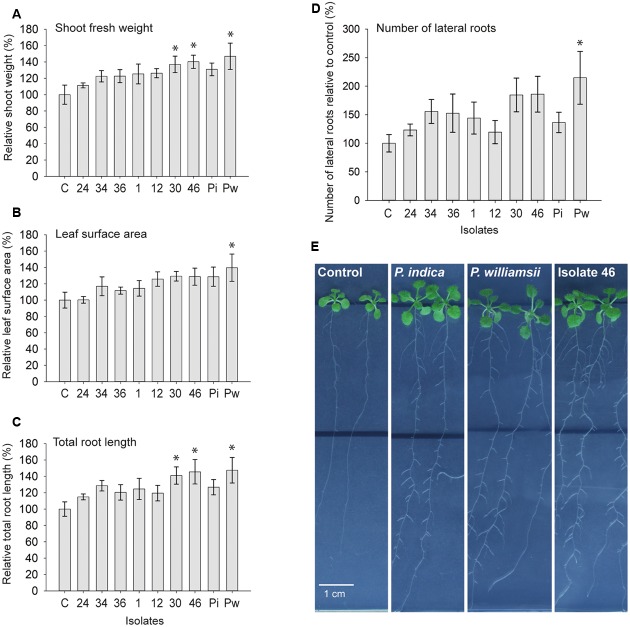
**Growth promotion effect of *Piriformospora* isolates on *Arabidopsis thaliana* (Col-0) in an *in vitro* assay. (A–D)** Five-day-old *Arabidopsis* seedlings growing on half-strength MS medium without sucrose were inoculated with 30,000 spores of each of the seven Congolese isolates (1, 12, 24, 30, 34, 36, and 46), and *P. indica* (Pi) and *P. williamsii* (Pw). Average shoot fresh weight of individual plants **(A)**, total leaf surface area per plate **(B)**, total length of main and lateral roots per plate **(C)**, and number of lateral roots per plate **(D)** were determined 9 days after inoculation. Error bars indicate standard errors on the mean of the ratios of the different treatments to the mock-inoculated control, based on five replicates. Asterisks indicate statistical significant differences compared to the control (one-sided Dunnett’s test α = 0.05). **(E)** Spore inoculation was done at a distance of 3.5 cm below the seedlings (black line) and plates were sealed with air-permeable plastic foil. A pronounced stimulation of lateral root formation and lateral root growth in the mature zone could be observed, as shown here for *P. indica*, *P. williamsii* and isolate 46. Full plates are included in Supplementary Figure S6.

In many reports the exceptional biological activities of *P. indica* have been praised and the organism has been described as a revolutionary PGP fungus (Waller et al., 2005; Achatz et al., 2010; Fakhro et al., 2010; Sun et al., 2010, 2014; Zamani et al., 2016). Stimulated by the phylogenetic relationship between the Congolese isolates and *P. indica*, their putative PGP potential was tested in an *in vitro* system with *Arabidopsis thaliana* as a host. Nine days after inoculation of 5-day-old seedlings with chlamydospores of the seven selected isolates and the two reference strains, shoot fresh weight per plant, leaf surface area per plate, total root length per plate (main plus lateral roots), and number of lateral roots per plate were determined. Compared to mock-inoculated controls, *P. indica* increased fresh shoot weight with 31%, which is in accordance with published results (Lahrmann et al., 2013; Banhara et al., 2015). In contrast to these reports, however, under our experimental conditions, *P. williamsii* also increased shoot yield by 47% (standard error of the difference between the means of *P. williamsii* and *P. indica* = 17.85%). Possibly, the use of different plant media and another inoculation method are at the basis of this discrepancy. Importantly, all Congolese isolates also positively affected shoot fresh weight with levels ranging between 11% for isolate 24 and 40% for isolate 46 (**Figure 7A**). Similar trends were observed for the leaf surface area (**Figure 7B**) and the total root length (**Figure 7C**). Whereas the morphology of the shoots was not affected by the fungal presence, the root architecture of the seedlings showed a strong response (**Figures 7D,E**; Supplementary Figure S6): all treatments resulted in a pronounced stimulation of lateral root formation with 20% (isolate 12) to 115% (*P. williamsii*) more lateral roots formed compared to the uninoculated controls (**Figure 7D**), contributing to a total root length increase of up to 47% (**Figure 7C**). Based on the analyzed parameters, it seems that isolates 30 and 46 and *P. williamsii* are outperforming the other five tested isolates and *P. indica*, which is reflected by the *P*-values of the one-sided Dunnett’s test for shoot weight, leaf surface area, total root length and number of lateral roots: respectively, 0.025, 0.195, 0.023, and 0.079 for isolate 30; 0.014, 0.207, 0.011, and 0.073 for isolate 46; and 0.003, 0.044, 0.007, and 0.012 for *P. williamsii*. Although several genes involved in early signaling events during the *P. indica*-host interaction have been identified (Oelmüller et al., 2005, 2009; Shahollari et al., 2007; Schäfer et al., 2009; Vadassery et al., 2009; Camehl et al., 2011; Jacobs et al., 2011; Lee et al., 2011), the exact mechanism by which this fungus impacts plant development remains to be uncovered. Hormone profiling and genome sequencing are anticipated to give a first glimpse on the PGP strategies of the Congolese isolates.

## Conclusion

With sudangrass-based trap systems 51 endophytic isolates of the genus *Piriformospora* were obtained from rhizospheric soil samples collected in the area of Kisangani. Appropriate storage of samples appeared to be imperative for efficient host colonization and isolation of the fungi, a finding that might facilitate future expansion of the Serendipitaceae. Based on morphological characteristics, ISSR profiles, and *ITS* and *TEF1α* sequences, seven closely related ‘genetic groups’ were distinguished among these isolates. The molecular data demonstrated that they do not belong to the same species as *P. indica*, but, together with *P. williamsii*, might constitute a species complex *Piriformospora* ‘*williamsii.*’ Just like the reference strains, the Congolese isolates stimulated biomass production and root growth of *Arabidopsis* seedlings in *in vitro* assays. In our experimental setup, some isolates performed better than the well-studied *P. indica*. With this collection of new *Piriformospora* isolates at hand, exciting challenges for future research will be to determine their exact taxonomic position, to unravel the mode of action behind their PGP capacity, and to assess their performance under adverse conditions. Moreover, the frequently observed coexistence with AMF might offer interesting opportunities to develop novel inocula. In conclusion, Kisangani soils seem to be a rich source of *Piriformospora* species that can be trapped with sudangrass as a highly conducive host. Their implementation as local inoculants in an ISFM approach will hopefully contribute to resolving serious issues in agriculture and horticulture in sub-Saharan Africa where plant production is impaired by diverse abiotic and biotic stresses.

## Author Contributions

JoV carried out the field and laboratory work. JoV, KA, GB, DV, and GH participated in the design of this study. JaV analyzed the *Arabidopsis* root data by the use of a dedicated software tool written in Python. AM and BD provided practical assistance during sampling in the Congolese fields. JoV, KA, DV, and GH were involved in generating the first drafts of the manuscript. JaV, GB, and PB critically revised the manuscript. All authors read and approved the final manuscript.

## Conflict of Interest Statement

The authors declare that the research was conducted in the absence of any commercial or financial relationships that could be construed as a potential conflict of interest.
