# Supplementary material for: Congolese Rhizospheric Soils as a Rich Source of New Plant Growth-Promoting Endophytic Piriformospora Isolates
Source: Front Microbiol. 2017 Feb 14;8:212. doi: 10.3389/fmicb.2017.00212 (PMC5306995; doi:10.3389/fmicb.2017.00212)
Supplement: Supplementary file 1 [file Data_Sheet_1.PDF]

Supplementary Materials and Methods

Congolese rhizospheric soils as a rich source of new plant growth-promoting endophytic *Piriformospora* isolates

Jolien Venneman[1], Kris Audenaert[1], Jan Verwaeren[1], Geert Baert[1], Pascal Boeckx[2], Adrien Moango Manga[3], Benoît Dhed'a Djailo[3], Danny Vereecke[1*†], and Geert Haesaert[1†]

*** Correspondence:** Danny Vereecke: danny.vereecke@ugent.be

[†]These authors have contributed equally to this work

Automated root phenotyping

Image acquisition

As part of the *in vitro* plant growth promotion assay with *Arabidopsis*, the leaf surface area, total root length (main plus lateral roots) and number of lateral roots (with a length of at least 2 mm) of the seedlings were determined per petri dish nine days after inoculation with chlamydospore suspensions. When *Arabidopsis* is grown vertically in plates, the roots do not penetrate the agar but grow mainly on the surface of the medium which allows high quality image acquisition of both shoot and root. Images of the seedlings were acquired using a Canon EOS 50D camera with a 60mm f/16 macro lens at 15 megapixels (72 dpi resolution).

Computation of traits

The leaf surface area was measured by hue-based thresholding using ASSESS 2.0 Image Analysis Software (Lamari, 2002). Despite the existence of a number of root analysis software tools (in particular EZ-Rhizo (Armengaud et al., 2009), RootGraph (Cai et al., 2015), BRAT (Slovak et al., 2014), ARIA (Pace et al., 2014)) that could potentially be used to compute the total root length and the number of lateral roots, none of these tools was found to be capable of analyzing the acquired images reliably[1]. Therefore, a dedicated software-tool was written in Python, relying on the *Skimage* library (van der Walt et al., 2014) for implementations of the most important image processing algorithms.

[1] The main raisons for their failure is probably related to some specific properties of the acquired images such as the limited width (in term of pixels) of the roots, the presence of both primary and secondary roots, the presence of artefacts and the presence of a variable background that prevents the use of simple thresholding techniques of the RGB or HSV image to discriminate between foreground (root) and background.

To be able to compute the total root length and the number of lateral roots, the acquired images were transformed into (skeletonized) binary images indicating the presence/absence of a root. The following routine was used to compute these images (steps 2-4 are illustrated in Supplementary Figure S1):

(1) *Cropping of the image*: as the position of the petri dishes was fixed within the images, the position of the dish could be used to locate the dish within the image and the images were cropped accordingly.
(2) *Ridge filtering*: the eigenvalues of the local Hessian matrix of an image provide information on the curvature of the image at a given pixel. The (opposite of the) main negative eigenvalue of the local Hessian matrix is computed for each pixel. The resulting image will amplify ridge-like patterns and eliminate or damp other patterns. To construct the Hessian matrix, second order derivatives were computed by repeatedly convolving the images with the first derivative of a Gaussian ($\sigma = 1.3$), in the horizontal and vertical direction.
(3) *Conversion to a binary image*: a threshold of 3.5×10^{-3} was used to obtain an initial binary image in which the roots are separated from the background. Closing and morphological reconstruction operations were used to connect broken ridges. Small, isolated objects (< 300 pixels) are considered artefacts and are removed from the resulting image using morphological operations.
(4) *Skeletonizing the image:* Skimages's skeletonization function was used to skeletonize the resulting image.

The number of foreground pixels of the skeleton (multiplied by the resolution) was used as the total root length (main plus lateral roots). Subsequently, the skeletonized image was used to determine the number of lateral roots. As the complete root system has a rather simple topology (one main root and a number of lateral roots) its analysis can be performed using simple operations. Root tips are pixels with exactly one neighbor, whereas branching points have at least three neighbors (using 8-connectivity). Using this information, individual root segments were identified and characterized as either being part of the main root (bounded by two branching points) or being a lateral root (bounded by one branching point and one tip pixel). Root segments belonging to the main root were removed. From the remaining segments the number of segments with a length of at least 2 mm was computed and reported as the number of lateral roots.

References

Armengaud, P., Zambaux, K., Hills, A., Sulpice, R., Pattison, R.J., Blatt, M.R. et al. (2009). EZ-Rhizo: integrated software for the fast and accurate measurement of root system architecture. *Plant J* 57, 945-956. doi: 10.1111/j.1365-313X.2008.03739.x

Cai, J., Zeng, Z., Connor, J.N., Huang, C.Y., Melino, V., Kumar, P. et al. (2015). RootGraph: a graphic optimization tool for automated image analysis of plant roots. *J Exp Bot* 66, 6551-6562. doi: 10.1093/jxb/erv359

Pace, J., Lee, N., Naik, H.S., Ganapathysubramanian, B., and Lübberstedt, T. (2014). Analysis of Maize (*Zea mays* L.) Seedling Roots with the High-Throughput Image Analysis Tool ARIA (Automatic Root Image Analysis). *PLoS One* 9: e108255. doi: 10.1371/journal.pone.0108255

Slovak, R., Göschl, C., Su, X., Shimotani, K., Shiina, T., and Busch, W. (2014). A Scalable Open-Source Pipeline for Large-Scale Root Phenotyping of *Arabidopsis*. *Plant Cell* 26, 2390-2403. doi: http://dx.doi.org/10.1105/tpc.114.124032

van der Walt, S., Schönberger, J.L., Nunez-Iglesias, J., Boulogne, F., Warner, J.D., Yager, N. et al. (2014). scikit-image: image processing in Python. *PeerJ* 2:e453. doi: 10.7717/peerj.453
