# Supplementary material for: Congolese Rhizospheric Soils as a Rich Source of New Plant Growth-Promoting Endophytic Piriformospora Isolates
Source: Front Microbiol. 2017 Feb 14;8:212. doi: 10.3389/fmicb.2017.00212 (PMC5306995; doi:10.3389/fmicb.2017.00212)
Supplement: Supplementary file 2 [file Data_Sheet_2.pdf]

Supplementary Tables and Figures

Congolese rhizospheric soils as a rich source of new plant growth-promoting endophytic *Piriformospora* isolates

Jolien Venneman[1], Kris Audenaert[1], Jan Verwaeren[1], Geert Baert[1], Pascal Boeckx[2], Adrien Moango Manga[3], Benoît Dhed'a Djailo[3], Danny Vereecke[1*†], and Geert Haesaert[1†]

*** Correspondence:** Danny Vereecke: danny.vereecke@ugent.be

[†]These authors have contributed equally to this work

Included in this file:

- **Table S1.** List of locations and associated plant systems that were sampled in this study.
- **Table S2.** Overview of media used for isolation and culturing of *Piriformospora*, and for *in vitro* assays with *Arabidopsis*.
- **Table S3**. List of primers used in this study for *Piriformospora* detection and sequence analysis.
- **Table S4.** List of ISSR primers used in this study for the preliminary screening with a subset of five *Piriformospora* isolates.
- **Figure S1.** Different steps in image processing to determine total root length and number of lateral roots of *in vitro Arabidopsis* seedlings.
- **Figure S2.** Ribosomal DNA PCR amplification and isolation of *Piriformospora* from chlamydospore-containing trap roots.
- **Figure S3.** Multiple sequence alignments of *Piriformospora ITS* (A) and *TEF1α* (B) sequences.
- **Figure S4.** *Piriformospora* mycelium in CM liquid culture.
- **Figure S5.** ISSR fingerprinting profiles of *Piriformospora* isolates.

  Figure S6. Growth promotion effect of *Piriformospora* isolates on *Arabidopsis thaliana* (Col-0) in an *in vitro* assay.

Table S1. **List of locations and associated plant systems that were sampled in this study.** Soil samples were incorporated in trap cultures with sudangrass as a host. After certain time points, trap roots were stained with Parker blue ink to score for absence (-), presence (+) or abundant presence (++) of *Piriformospora* (PIRI) and arbuscular mycorrhizal fungi (AMF). The trap cultures set up after the first sampling round in Kisangani (A-L) were evaluated after six weeks; the traps from the second sampling (BAN 1-13, IT 1-2, MAN 1-5, MAS 1-10, SS 1-7, YOK 1-2) were assessed six, eight and ten months after installation of the first trap, and six weeks after installation of the secondary trap. For six traps, indicated by an asterisk (*), *Piriformospora* cultures could be obtained from surface sterilized sudangrass roots: seven isolates from MAS 2 (isolates 1-7), sixteen from SS 3 (isolates 8-23), two from MAN 2 (isolates 24-25), five from MAN 3 (isolates 26-30), eight from MAS 6 (isolates 31-38), and thirteen from MAS 9 (isolates 39-51).

Sample code	Location of sampling (latitude N, longitude E in decimal degrees)	Plant system sampled	Presence of *Piriformospora* in trap culture	Presence of AMF in trap culture
**A**	Simi-Simi, 14 km from Kisangani (N: 0.55332; E: 25.09207)	Fern	-	-
B	5 km from Kisangani, axis to village of Simi-Simi (N: 0.51435; E: 25.16081)	*Pueraria javanica* & other herbaceous plants	-	+
C	18 km from Kisangani, axis to city of Ituri (N: 0.47267; E: 25.33470)	Soybean	-	-
D	Mandombe, 11 km from Kisangani, axis to city of Ituri (N: 0.49024; E: 25.28562)	Pineapple	+	+
E	Mandombe (N: 0.48738; E: 25.28308)	Banana	-	+
F	Mandombe (N: 0.49091; E: 25.28521)	Poaceae herbaceous plant	-	-
G	Mandombe (N: 0.48624; E: 25.28425)	*Pueraria javanica* & other herbaceous plants	-	-
H	Mandombe (N: 0.49003; E: 25.28296)	*Pueraria javanica* in oil palm plantation	-	+
I	11 km from Kisangani, axis to village of Masako (N: 0.58789; E: 25.25166)	*Pueraria javanica* & other herbaceous plants	-	+
J	Masako, 14 km from Kisangani (N: 0.61089; E: 25.26085)	Poaceae herbaceous plant	-	-
K	Masako (N: 0.60227; E: 25.26408)	*Chromolaena odorata*	-	-
L	Masako (N:0.60097; E: 25.26599)	Pineapple	-	+

**BAN 1**	12 km from Kisangani, axis to city of Banalia (N: 0.60694; E: 25.16927)	Poaceae herbaceous plant	+	++
BAN 2	12 km from Kisangani, axis to city of Banalia	*Fabaceae* herbaceous plant	-	-
BAN 3	12 km from Kisangani, axis to city of Banalia	*Chromolaena odorata*	-	-
BAN 4	12 km from Kisangani, axis to city of Banalia	*Pueraria javanica* & other herbaceous plants	-	+
BAN 5	12 km from Kisangani, axis to city of Banalia	Pineapple	+	+
BAN 6	12 km from Kisangani, axis to city of Banalia	Banana	-	+
BAN 7	15 km from Kisangani, axis to city of Banalia (N: 0.63835; E: 25.18911)	Poaceae herbaceous plant	+	++
BAN 8	15 km from Kisangani, axis to city of Banalia	Sugar cane	-	-
BAN 9	15 km from Kisangani, axis to city of Banalia	*Chromolaena odorata*	-	-
BAN 10	15 km from Kisangani, axis to city of Banalia	Banana	-	-
BAN 11	15 km from Kisangani, axis to city of Banalia	Pineapple	-	-
BAN 12	15 km from Kisangani, axis to city of Banalia	Maize, intercropped with cassava	+	+
BAN 13	10 km from Kisangani, axis to city of Banalia	Maize, intercropped with cassava & groundnut	-	-
**IT 1**	8 km from Kisangani, axis to city of Ituri (N: 0.51726; E: 25.26140)	Maize, intercropped with cassava & taro	-	-
IT 2	18 km from Kisangani, axis to city of Ituri (cf. location of sample C)	Maize	+	-
**MAN 1**	Mandombe, 11 km from Kisangani, axis to city of Ituri (cf. location of samples D-H)	*Pueraria javanica*	+	+
MAN 2*	Mandombe	Pineapple	+	-
MAN 3*	Mandombe	Banana	++	++
MAN 4	Mandombe	Poaceae herbaceous plant	+	++
MAN 5	Mandombe	*Chromolaena odorata*	+	-
**MAS 1**	Masako, 14 km from Kisangani (cf. location of samples J-L)	Wild cassava & herbaceous plants	-	-

MAS 2*	Masako	Sugar cane	++	++
MAS 3	Masako	Pineapple	-	-
MAS 4	Masako	Banana	-	+
MAS 5	Masako	*Chromolaena odorata*	-	-
MAS 6*	Masako	Poaceae & other herbaceous plants	++	++
MAS 7	Masako	Fern	-	-
MAS 8	Masako (cf. location of sample L)	Maize, intercropped with banana & pineapple	+	+
MAS 9*	Masako (cf. location of sample I)	Maize, intercropped with *Vigna*	++	-
MAS 10	7 km from Kisangani, axis to village of Masako (N: 0.56126; E: 25.22119)	Maize, intercropped with cassava	+	-
**SS 1**	Simi-Simi, 14 km from Kisangani (cf. location of sample A)	Pineapple	-	-
SS 2	Simi-Simi	Poaceae herbaceous plant	-	-
SS 3*	Simi-Simi	Fern	++	-
SS 4	Simi-Simi	*Chromolaena odorata*	-	+
SS 5	Simi-Simi	Banana	-	-
SS 6	Simi-Simi	Maize, intercropped with cassava	-	++
SS 7	5 km from Kisangani, axis to village of Simi-Simi (cf. location of sample B)	Maize, intercropped with cassava & groundnut	+	+
**YOK 1**	Yoko, 32 km from Kisangani (N: 0.29358; E: 25.28611)	Maize, intercropped with rice	+	-
YOK 2	Yoko, 32 km from Kisangani (N: 0.28694; E: 25.28139)	Banana	-	-

Table S2. Overview of media used for isolation and culturing of *Piriformospora*, and for *in vitro* assays with *Arabidopsis*. CM, MEA, MYP, MMN, PDA and water agar were used for isolation of *Piriformospora*; only PDA was used for continuous subculturing and harvest of spores. Mycelium for DNA extraction was grown in PDB and *Arabidopsis* experiments were done on ½ MS without sucrose.

Medium	Composition
Complex medium (CM; modified *Aspergillus* medium)	Pham et al., 2004
Malt extract agar (MEA)	CM0059, Oxoid, Erembodegem, Belgium
Malt yeast peptone (MYP)	7.0 g/l malt extract, 1.0 g/l peptone, 0.5 g/l yeast extract and 15 g/l agar-agar, pH 5.8
Modified Melin-Norkrans (MMN)	Johnson et al., 1957
Murashige and Skoog (MS), half-strength without sucrose	0.5 g l/l MES monohydrate, 0.1 g/l myo-inositol, 2.2 g/l MS salts including vitamins and 8.0 g/l plant agar, pH 5.7
Potato dextrose agar (PDA)<br>Potato dextrose broth (PDB)	CM0139, Oxoid, Erembodegem, Belgium<br>P6685, Sigma-Aldrich, Diegem, Belgium
Water agar 1.5 %	15 g/l agar-agar

Table S3. List of primers used in this study for *Piriformospora* detection and sequence analysis.

Primer name	Primer sequence 5'→3'	Reference
NSSeb1	CTTCTTAGAGGGACTGTCAGGA	Weiß et al., 2011
NLSeb2R	GCCCACTAGAAACTCTCACC	Weiß et al., 2011
ITS1F	CTTGGTCATTTAGAGGAAGTAA	Gardes and Bruns, 1993
ITS2	GCTGCGTTCTTCATCGATGC	White et al., 1990
ITS3	GCATCGATGAAGAACGCAGC	White et al., 1990
ITS4	TCCTCCGCTTATTGATATGC	White et al., 1990
NL1	GCATATCAATAAGCGGAGGAAAAG	O'Donnell, 1993
NL4	GGTCCGTGTTTCAAGACGG	O'Donnell, 1993
NLB4	GGATTCTCACCCTCTATGAC	Martin and Rygiewicz, 2005
EF1-983F	GCYCCYGGHCAYCGTGAYTTYAT	Rehner and Buckley, 2005
EF1-2218R	ATGACACCRACRGCRACRGTYTG	Rehner and Buckley, 2005
1567R	ACHGTRCCRATACCACCSATCTT	Rehner and Buckley, 2005
1577F	CARGAYGTBTACAAGATYGGTGG	Rehner and Buckley, 2005

Table S4. List of ISSR primers used in this study for the preliminary screening with a subset of five *Piriformospora* isolates. Primers retained for final analysis with all isolates are indicated (*).

Primer sequence	Number of clear amplified bands	Polymorphism (%)	Reference
(AC)8C*	9	89	Consolo et al., 2015; Palmero et al., 2014
(AC)8YG*	15	71	Palmero et al., 2014
(AG)8C	10	40	Chadha and Gopalakrishna, 2009; Estrada et al., 2007
(AG)8G	11	55	Bayraktar et al., 2008; Chadha and Gopalakrishna, 2009
(GA)8YC*	17	82	Bayraktar et al., 2008
(GA)8YG*	12	75	Estrada et al., 2007
(AAC)6	1	0	Pintye et al., 2012
(AAG)6	6	50	Pintye et al., 2012
(CAG)5	12	50	Boss et al., 2007
(CTC)6	11	45	Palmero et al., 2014
(GTG)5	12	58	Curlevski et al., 2009
(TC)8RG	/	/	Consolo et al., 2015
HVH(TG)7	6	67	Chadha and Gopalakrishna, 2009
DDB(CCA)5	12	58	Curlevski et al., 2009
(GACA)4	13	62	Curlevski et al., 2009; Estrada et al., 2007

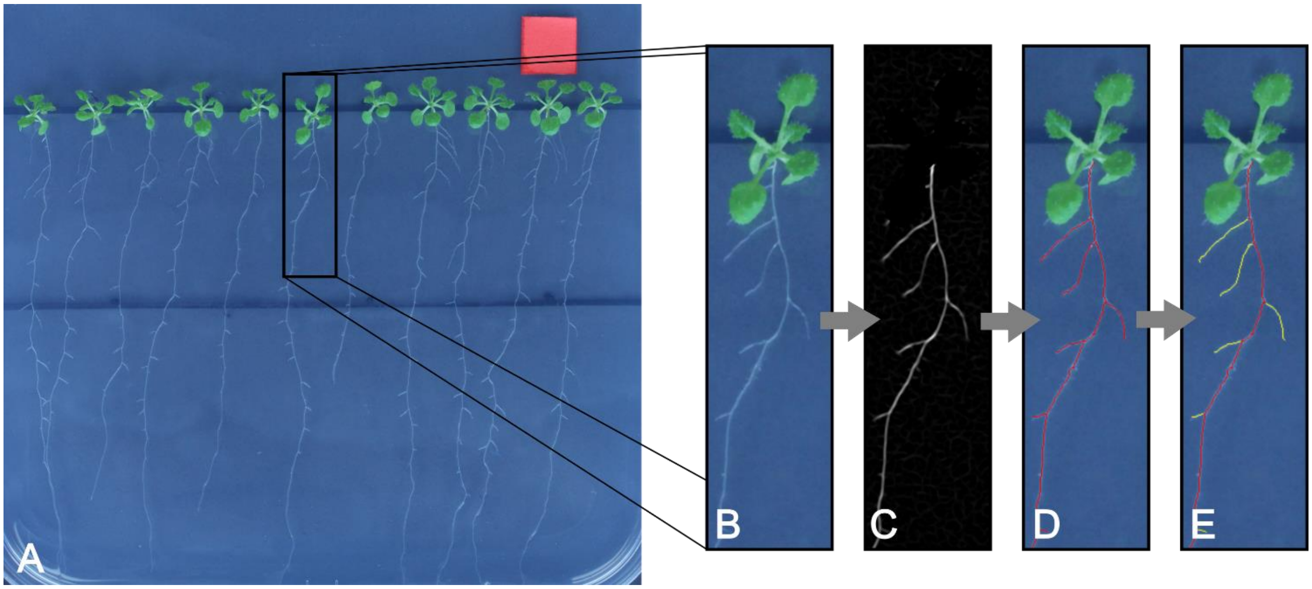

Figure S1. Different steps in image processing to determine total root length and number of lateral roots of *in vitro Arabidopsis* seedlings. (A) Original image. (B) Enlarged part of the original image. (C) Image after ridge filtering. (D) Thresholded and skeletonized root image superimposed (in red) on the original image. (E) Result obtained when differentiating between the main root (red) and lateral roots (yellow).

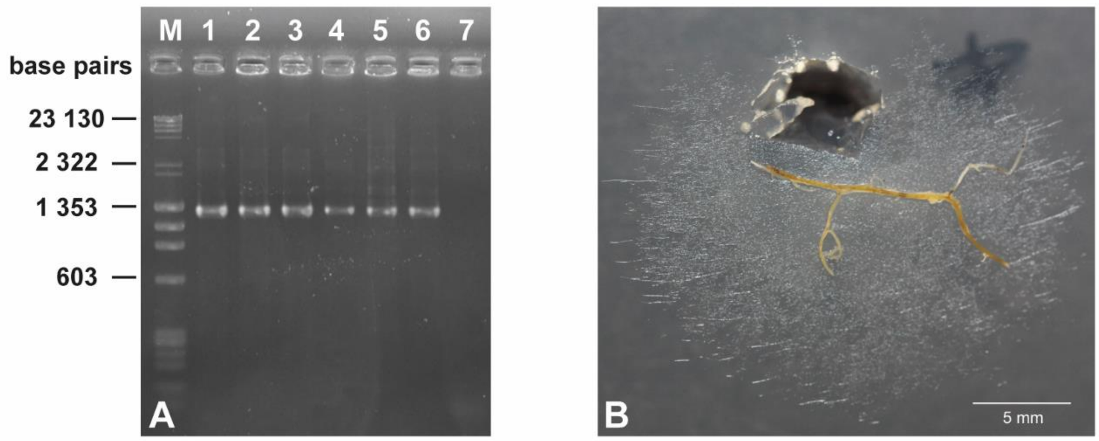

Figure S2. Ribosomal DNA PCR amplification and isolation of *Piriformospora* from chlamydospore-containing trap roots. (**A**) Nested PCR with universal fungal primers ITS1F and NL4 after a Sebacinales-specific PCR with primers NSSeb1 and NLSeb2R. The ~1,250 bp DNA fragment comprises the internal transcribed spacer 1 (ITS1), the 5.8S subunit, ITS2, and part of the large subunit (LSU; 28S) of the ribosomal DNA. 1, sorghum root from trap MAN 2; 2, sorghum root from trap MAN 3; 3, sorghum root from trap MAS 2; 4, sorghum root from trap MAS 9; 5, sorghum root from trap SS 3; 6, maize root from trap D; 7, non-template control; M, broad range DNA marker (Lambda HindIII + φX174 HaeIII digest). (**B**) Surface sterilized 1-to-2-cm root piece of sudangrass trap root placed on PDA medium and incubated at 22°C. Four to eight days later, *Piriformospora* outgrowth became visible and was transferred to a new PDA plate.

A) Multiple sequence alignment of internal transcribed spacer 1 (ITS1), 5.8S subunit and ITS2

```
Isolate_1          1 TTCGATTCGGTCT-----TCTGTGCTGGTGGCAA--CACATGTGCACG---TTGATCGTA
Isolate_46         1 TTCGATTCGGTCT-----TCTGTGCTGGTGGCAA--CACATGTGCACG---TTGATCGTA
Isolate_12         1 TTCGATTCGGTCT-----TCTGTGCTGGTGGCAA--CACATGTGCACG---TTGATCGTA
root_from_trapD    1 TTCGATTCGGTCT-----TCTGTGCTGGTGGCAA--CACATGTGCACG---TTGATCGTA
Isolate_30         1 TTCGATTCGGTCT-----TCTGTGCTGGTGGCAA--CACATGTGCACG---TTGATCGTA
Isolate_24         1 TTCGATTCGGTCT-----TCTGTGCTGGTGGCAA--CACATGTGCACG---TTGATCGTA
Isolate_34         1 TTCGATTCGGTCT-----TCTGTGCTGGTGGCAA--CACATGTGCACG---TTGATCGTA
Isolate_36         1 TTCGATTCGGTCT-----TCTGTGCTGGTGGCAA--CACATGTGCACG---TTGATCGTA
P.williamsii       1 TTCGATTCGGTCT-----TCTGTGCTGGTGGCAA--CACATGTGCACG---TTGATCGTA
P.indica           1 TTCGATTCGGTCT-----TCTGTGCTGGTGGCAA--CACATGTGCACG---TCGATCGTA
KF061284_P.indi    1 TTCGATTCGGTCT-----TCTGTGCTGGTGGCAA--CACATGTGCACG---TCGATCGTA
KF061285_S.herb    1 TTCAAAGTCGGCCAAAGGTTGTTGCTGGTGATTATTCACATGTGCACGCCTTTGGTCGCA
EU626002_S.verm    1 TACAAAGTCGGCC--TTGCTGTTGCTGGTGGAAA--CACATGTGCACGC--TCGGTCGCA

Isolate_1         51 ATTC--CATTCTCCTTTCAATCGTCCAAGTGTGTTCGGTCGGCCTTTTGG-----GTCGA
Isolate_46        51 ATTC--CATTCTCCTTTCAATCGTCCAAGTGTGTTCGGTCGGCCTTTTGG-----GTCGA
Isolate_12        51 ATTC--CATTCTCCTTTCAATCGTCCAAGTGTGTTCGGTCGGCCTTTTGG-----GTCGA
root_from_trapD   51 ATTC--CATTCTCCTTTCAATCGTCCAAGTGTGTTCGGTCGGCCTTTTGG-----GTCGA
Isolate_30        51 ATTC--CATTCTCCTTTCAATCGTCCAAGTGTGTTCGGTCGGCCTTTTGG-----GTCGA
Isolate_24        51 ATTC--CATTCTCCTTTCAATCGTCCAAGTGTGTTCGGTCGGCCTTTTGG-----GTCGA
Isolate_34        51 ATTC--CATTCTCCTTTCAATCGTCCAAGTGTGTTCAGTCGGCCTTTTGG-----GTCGA
Isolate_36        51 ATTC--CATTCTCCTTTCAATCGTCCAAGTGTGTTCAGTCGGCCTTTTGG-----GTCGA
P.williamsii      51 ATTC--CATTCTCCTTTCAATCGTCCAAGTGTGTTCGGTYGGCCTTTTGG-----GTCGA
P.indica          51 ATTC--CATCCTCCTTTCAATTGTCCAAGTGCGTTCGGTCGGCC-TCTGG-----GTCGA
KF061284_P.indi   51 ATTC--CATCCTCCTTTCAATTGTCCAAGTGCGTTCGGTCGGCC-TCTGG-----GTCGA
KF061285_S.herb   61 AATCCACACACACCTGT-----------GCATCTATGACTCGAG-GTTGT-----AATGA
EU626002_S.verm   55 AATC--CACACACCTGT-----------GCATCTATGACCCGAG-GTTGTCGTCGTATGA

Isolate_1        104 AGAAGCGTGGCCGTCAGGCTCGTTTCGTTCYGAGCGCACATTGGTTATTTCACCCACTCT
Isolate_46       104 AGAAGCGTGGCCGTCAGGCTCGTTTCGTTCYGAGCGCACATTGGTTATTTCACCCACTCT
Isolate_12       104 AGAAGCGTGGCCGTCAGGCTCGTTTCGTTCTGAGCGCACATTGGTTATTTCACCCACTCT
root_from_trapD  104 AGAAGCGTGGCCGTCAGGCTCGTTTCGTTCTGAGCGCACATTGGTTATTTCACCCACTCT
Isolate_30       104 AGAAGCGTGGCCGTCAGGCTCGTTTCGTTCYGAGCGCACATTGGTTATTTCACCCACTCT
Isolate_24       104 AGAAGCGTGGCCGTCAGGCTCGTTTCGTTCYGAGCGCACATTGGTTATTTCACCCACTCT
Isolate_34       104 AGAAGCATGGCCGTCAGGCTCGTTTCGTTCCGAGCGCACATTGGTTATTTCACCCACTCT
Isolate_36       104 AGAAGCATGGCCGTCAGGCTCGTTTCGTTCCGAGCGCACATTGGTTATTTCACCCACTCT
P.williamsii     104 AGAAGCGTGGCCGTCAGGCTCGTTTCSTTCTGAGCGCACATTGGTTATTTCACCCACTCT
P.indica         103 AGAAGCGTGGCTGCCAGGCTCGTTTCGTTCTGAATGCACATTGGTTATTTCACCCACTCT
KF061284_P.indi  103 AGAAGCGTGGCTGCCAGGCTCGTTTCGTTCTGAATGCACATTGGTTATTTCACCCACTCT
KF061285_S.herb  104 CTGAGAGTAACTTTTATACACTCTTGTATGTAATGGAATGT---------------CTTT
EU626002_S.verm  101 CCGAGGGTAAAATTTACACACTCTTGAATGTAATGGAATGT-------CTCTTGTGCATA

Isolate_1        164 GGGTACTCGAAACAAAGAAAACAAATCATATAAATTAAACAACTTTCAACAACGGATCTC
Isolate_46       164 GGGTACTCGAAACAAAGAAAACAAATCATATAAATTAAACAACTTTCAACAACGGATCTC
Isolate_12       164 GGGTACTCGAAACAAAGAAAACAAATCATATAAATTAAACAACTTTCAACAACGGATCTC
root_from_trapD  164 GGGTACTCGAAACAAAGAAAACAAATCATATAAATTAAACAACTTTCAACAACGGATCTC
Isolate_30       164 GGGTACTCGAAACAAAGAAAAMAAATCATATAAATTAAACAACTTTCAACAACGGATCTC
Isolate_24       164 GGGTACTCGAAACAAAGAAAACAAATCATATAAATTAAACAACTTTCAACAACGGATCTC
Isolate_34       164 GGGTACTCGAAACAAAGAAAACAAATCATATAAATTAAACAACTTTCAACAACGGATCTC
Isolate_36       164 GGGTACTCGAAACAAAGAAAACAAATCATATAAATTAAACAACTTTCAACAACGGATCTC
P.williamsii     164 GGGTACTCGAAACAAAGAAAAMAANNNNNNNNNNNNNNNNNNNNNNNNNNNNNNNNNNNN
P.indica         163 GGGTACTAGAAACAAAGAAA--TATTCATATAAACTATACAACTTTCAACAACGGATCTC
KF061284_P.indi  163 GGGTACTAGAAACAAAGAAA--TATTCATATAAACTATACAACTTTCAACAACGGATCTC
KF061285_S.herb  149 TTGTGCTATAA----------------TGTACAATATAAACAACTTTCAACAACGGATCTC
EU626002_S.verm  154 ATGTAC---------------------------AATTAAACAACTTTCAACAACGGATCTC

Isolate_1        224 TTGGCTCTCGCATCGATGAAGAACGCAGCGAAATGCGATAAGTAATGTGAATTGCAGAAT
Isolate_46       224 TTGGCTCTCGCATCGATGAAGAACGCAGCGAAATGCGATAAGTAATGTGAATTGCAGAAT
Isolate_12       224 TTGGCTCTCGCATCGATGAAGAACGCAGCGAAATGCGATAAGTAATGTGAATTGCAGAAT
root_from_trapD  224 TTGGCTCTCGCATCGATGAAGAACGCAGCGAAATGCGATAAGTAATGTGAATTGCAGAAT
Isolate_30       224 TTGGCTCTCGCATCGATGAAGAACGCAGCGAAATGCGATAAGTAATGTGAATTGCAGAAT
Isolate_24       224 TTGGCTCTCGCATCGATGAAGAACGCAGCGAAATGCGATAAGTAATGTGAATTGCAGAAT
Isolate_34       224 TTGGCTCTCGCATCGATGAAGAACGCAGCGAAATGCGATAAGTAATGTGAATTGCAGAAT
Isolate_36       224 TTGGCTCTCGCATCGATGAAGAACGCAGCGAAATGCGATAAGTAATGTGAATTGCAGAAT
P.williamsii     224 NNNNNNNNNNNNNNNNNNNNNNNNNNNNNNNNNNNNNNNNNNNNNNNNNNNNNNNNNNNN
P.indica         221 TTGGCTCTCGCATCGATGAAGAACGCAGCGAAATGCGATAAGTAATGTGAATTGCAGAAT
KF061284_P.indi  221 TTGGCTCTCGCATCGATGAAGAACGCAGCGAAATGCGATAAGTAATGTGAATTGCAGAAT
KF061285_S.herb  194 TTGGCTCTCGCATCGATGAAGAACGCAGCGAAATGCGATAAGTAATGTGAATTGCAGAAT
EU626002_S.verm  188 TTGGCTCTCGCATCGATGAAGAACGCAGCGAAATGCGATAAGTAATGTGAATTGCAGAAT
```

```
Isolate_1          284 TCAGTGAATCATCGAATCTTTGAACGCACCTTGCACCCTTTGGTATTCCGAAGGGTACGC
Isolate_46         284 TCAGTGAATCATCGAATCTTTGAACGCACCTTGCACCCTTTGGTATTCCGAAGGGTACGC
Isolate_12         284 TCAGTGAATCATCGAATCTTTGAACGCACCTTGCACCCTTTGGTATTCCGAAGGGTACGC
root_from_trapD    284 TCAGTGAATCATCGAATCTTTGAACGCACCTTGCACCCTTTGGTATTCCGAAGGGTACGC
Isolate_30         284 TCAGTGAATCATCGAATCTTTGAACGCACCTTGCACCCTTTGGTATTCCGAAGGGTACGC
Isolate_24         284 TCAGTGAATCATCGAATCTTTGAACGCACCTTGCACCCTTTGGTATTCCGAAGGGTACGC
Isolate_34         284 TCAGTGAATCATCGAATCTTTGAACGCACCTTGCACCCTTTGGTATTCCGAAGGGTACGC
Isolate_36         284 TCAGTGAATCATCGAATCTTTGAACGCACCTTGCACCCTTTGGTATTCCGAAGGGTACGC
P.williamsii       284 NNNNNNNNNNNNNNNNNNNNNNNNCGCACCTTGCACCCTTTGGTATTCCGAAGGGTACGC
P.indica           281 TCAGTGAATCATCGAATCTTTGAACGCACCTTGCACCCTTTGGTATTCCGAAGGGTACGC
KF061284_P.indi    281 TCAGTGAATCATCGAATCTTTGAACGCACCTTGCACCCTTTGGTATTCCGAAGGGTACGC
KF061285_S.herb    254 TCAGTGAATCATCGAATCTTTGAACGCACCTTGCACCCTTTGGTATTCCGAAGGGTACGC
EU626002_S.verm    248 TCAGTGAATCATCGAATCTTTGAACGCACCTTGCACCCTTTGGTATTCCGAAGGGTACGC

Isolate_1          344 CCGTTTGAGTGTCGTTGTACTCTCAATCCTAC-ACTTTATTGTCGTATGGAT-TGGACTT
Isolate_46         344 CCGTTTGAGTGTCGTTGTACTCTCAATCCTAC-ACTTTATTGTCGTATGGAT-TGGACTT
Isolate_12         344 CCGTTTGAGTGTCGTTGTACTCTCAATCCTAC-ACTTTATTGTCGTATGGAT-TGGACTT
root_from_trapD    344 CCGTTTGAGTGTCGTTGTACTCTCAATCCTAC-ACTTTATTGTCGTATGGAT-TGGACTT
Isolate_30         344 CCGTTTGAGTGTCGTTGTACTCTCAATCCTAC-ACTTTATTGTCGTATGGAT-TGGACTT
Isolate_24         344 CCGTTTGAGTGTCGTTGTACTCTCAATCCTAC-ACTTTATTGTCGTATGGAT-TGGACTT
Isolate_34         344 CCGTTTGAGTGTCGTTGTACTCTCAATCCTAC-ACTTTATTGTCGTATGGAT-TGGACTT
Isolate_36         344 CCGTTTGAGTGTCGTTGTACTCTCAATCCTAC-ACTTTATTGTCGTATGGAT-TGGACTT
P.williamsii       344 CCGTTTGAGTGTCGTTGTACTCTCAATCCTAC-ACTTTATTGTCGTATGGAT-TGGACTT
P.indica           341 CCGTTTGAGTGTCGTTGTACTCTCAATCCTAC-AATTT-TTGTTGTACGGAT-TGGACTT
KF061284_P.indi    341 CCGTTTGAGTGTCGTTGTACTCTCAATCCTAC-AATTT-TTGTTGTACGGAT-TGGACTT
KF061285_S.herb    314 CCGTTTGAGTGTCATTGTAATCTCACTTCTACAACTTTATTGTTGTTTGAATGTGGACTT
EU626002_S.verm    308 CCGTTTGAGCGTCATTGTAATCTCACTTCTACCACTTTGTTGTTGTA-GAATGTGGACTT

Isolate_1          402 GGGTC-CTGTTGCGYT-CTGCAACTGATCCGAAATGTATCAGCGTGTGTCCGT-------
Isolate_46         402 GGGTC-CTGTTGCGYT-CTGCAACTGATCCGAAATGTATCAGCGTGTGTCCGT-------
Isolate_12         402 GGGTC-CTGTTGCGCT-CTGCAACTGATCCGAAATGTATCAGCGTGTGTCCGT-------
root_from_trapD    402 GGGTC-CTGTTGCGYT-CTGCAACTGATCCGAAATGTATCAGCGTGTGTCCGT-------
Isolate_30         402 GGGTC-CTGTTGCGYT-CTGCAACTGATCCGAAATGTATCAGCGTGTGTCCGT-------
Isolate_24         402 GGGTC-CTGTTGCGCT-CTGCAACTGATCCGAAATGTATCAGCSTGTGTCCGT-------
Isolate_34         402 GGGTC-CTGTTGCGCT-CTGCAACTGATCCGAAATGTATCAGCGTGTGTCCGT-------
Isolate_36         402 GGGTC-CTGTTGCGCT-CTGCAACTGATCCGAAATGTATCAGCGTGTGTCCGT-------
P.williamsii       402 GGGTC-CTGTTGCGTT-CTGCAACTGATCCGAAATGTATCAGCGTGTGTCCGT-------
P.indica           398 GGGTC-CTGTTGCGCT-CTGCAACTGATCCGAAATGTATCAGCGTGTGTCCGT-------
KF061284_P.indi    398 GGGTC-CTGTTGCGCT-CTGCAACTGATCCGAAATGTATCAGCGTGTGTCCGT-------
KF061285_S.herb    374 GGATG-TTGCCGTTTTATCACGGCTCRTCTTAAATGCCTGAGTGTACCCTGTTTYGCAGC
EU626002_S.verm    367 GGACGTCTGCCGTGTCACAACGGCTCGTCTTAAATGCCTGAGTGTACCCCGCTTTGCGGC

Isolate_1          453 -----CGTGACGATGAACCGATTGTTAAGTTTCATCGG--------GCTCGCTTTG----
Isolate_46         453 -----CGTGACGATGAACCGATTGTTAAGTTTCATCGG--------GCTCGCTTTG----
Isolate_12         453 -----CGTGACGATGAACCGATTGTTAAGTTTCATCGG--------GCTCGCTTTG----
root_from_trapD    453 -----CGTGACGATGAACCGATTGTTAAGTTTCATCGG--------GCTCGCTTTG----
Isolate_30         453 -----CGTGACGATGAACCGATTGTTAAGTTTCATCGG--------GCTCGCTTTG----
Isolate_24         453 -----CGTGACGATGAACCGATTGTTAAGTTTCATCGG--------GCTCGCTTTG----
Isolate_34         453 -----CGTGACGATGAACCGATTGTTAAGTTTCATCGG--------GCTCGCTTTG----
Isolate_36         453 -----CGTGACGATGAACCGATTGTTAAGTTTCATCGG--------GCTCGCTTTG----
P.williamsii       453 -----CGTGACGATGAACCGATTGTTAAGTTTCATCGG--------GCTCGCTTTG----
P.indica           449 -----CGTGACGATGAACCGATTGTTAAGTTTCATCGG--------GCTCGCTTTG----
KF061284_P.indi    449 -----CGTGACGATGAACCGATTGTTAAGTTTCATCGG--------GCTCGCTTTG----
KF061285_S.herb    433 GTATCCAGTGTAATAAACATTTCACTAGAGTTGGTCGAAAGACCGCGTTGACTATA----
EU626002_S.verm    427 GTATTCGGTGTGATAAACATTTCACCGGAGTTTGGTCTCTGACCGCGCTTTTAGCAATTG

                                                                  *
Isolate_1          496 -TCGAGGTCGTCT-GACGAATCCACATGCCTAACCCCNNNNN
Isolate_46         496 -TCGAGGTCGTCT-GACGAATCCACATGCCTAACCCCNNNNN
Isolate_12         496 -TCGAGGTCGTCT-GACGAATCCACATGCCTAACCCCCTATT
root_from_trapD    496 -TCGAGGTCGTCT-GACGAATCCACATGCCTAACCCCCTATT
Isolate_30         496 -TCGAGGTCGTCT-GACGAATCCACATGCCTAACCCCNNNNN
Isolate_24         496 -TCGAGGTCGTCT-GACGAATCCACATGCCTAACCCCNNNNN
Isolate_34         496 -TCGAGGTCGTCT-GACGAATCCACATGCCTAACCCCTATTA
Isolate_36         496 -TCGAGGTCGTCT-GACGAATCCACATGCCTAACCCCTATTA
P.williamsii       496 -TCGAGGTCGTCT-GACGAATCCACATGCCTAACCCCNNNNN
P.indica           492 -TCGAGGTCGTCT-GACGAA-ATGCATGCCTAACCCTCCTAT
KF061284_P.indi    492 -TCGAGGTCGTCT-GACGAA-ATGCATGCCTAACCCTCCTAT
KF061285_S.herb    489 -TGGGCTCTATGC-TTCAAA-CYGTCTGCCATGTGGACAATT
EU626002_S.verm    487 GTGGGCTCTATGCTTTCTAA-CCGTCT---TTATAAGACAAT
```

B) Multiple sequence alignment of translation elongation factor 1-α (TEF1α)

```
Isolate_1          1 TTGGCTTGCTCGGGATGAGCTTGACAATGGCRGCGTCACCAGACTTGACAAACTTGGGTG
Isolate_24         1 TTGGCTTGCTCGGGATGAGCTTGACAATGGCRGCGTCACCAGACTTGACAAACTTGGGTG
Isolate_46         1 TTGGCTTGCTCGGGATGAGCTTGACAATGGCRGCGTCACCAGACTTGACAAACTTGGGTG
Isolate_30         1 TTGGCTTGCTCGGGATGAGCTTGACAATGGCRGCGTCACCAGACTTGACAAACTTGGGTG
Isolate_34         1 TTGGCTTGCTCGGGATGAGCTTGACAATGGCGGCGTCACCAGACTTGACAAACTTGGGTG
Isolate_36         1 TTGGCTTGCTCGGGATGAGCTTGACAATGGCGGCGTCACCAGACTTGACAAACTTGGGTG
Isolate_12         1 TTGGCTTGCTCGGGATGAGCTTGACAATGGCAGCGTCACCAGACTTGACAAACTTGGGTG
P.williamsii       1 TTGGCTTGCTCGGGATGAGCTTGACAATGGCAGCGTCACCAGACTTGACAAACTTGGGTG
JN211110_P.will    1 TTGGCTTGCTCGGGATGAGCTTGACAATGGCAGCGTCACCAGACTTGACAAACTTGGGTG
P.indica           1 TTGGCTTGCTCGGGACGAGCTTGACAATGGCAGCGTCACCAGACTTGACAAACTTGGGGG
AJ249911_P.indi    1 TTGGCTTGCTCGGGACGAGCTTGACAATGGCAGCGTCACCAGACTTGACAAACTTGGGGG
JN211112_S.verm    1 TTGGCTTGCTTGGGATGAGTTTGACAATGGCGGCATCACCCATCTTGACAAACTTGGGGG

Isolate_1         61 CGGCTTCCATGGTCTTGCCAGTTCGGCGATCAATCTTTTCAATGAGCTCGGCGAACTTGC
Isolate_24        61 CGGCTTCCATGGTCTTGCCAGTTCGGCGATCAATCTTTTCAATGAGCTCGGCGAACTTGC
Isolate_46        61 CGGCTTCCATGGTCTTGCCAGTTCGGCGATCAATCTTTTCAATGAGCTCGGCGAACTTGC
Isolate_30        61 CGGCTTCCATGGTCTTGCCAGTTCGGCGATCAATCTTTTCAATGAGCTCGGCGAACTTGC
Isolate_34        61 CGGCTTCCATGGTCTTGCCAGTTCGGCGATCAATCTTTTCAATGAGCTCGGCGAACTTGC
Isolate_36        61 CGGCTTCCATGGTCTTGCCAGTTCGGCGATCAATCTTTTCAATGAGCTCGGCGAACTTGC
Isolate_12        61 CGGCTTCCATGGTCTTGCCAGTTCGGCGATCAATCTTTTCAATGAGCTCGGCGAACTTGC
P.williamsii      61 CGGCTTCCATGGTCTTGCCAGTTCGGCGATCAATCTTTTCAATGAGCTCGGCGAACTTGC
JN211110_P.will   61 CGGCTTCCATGGTCTTGCCAGTTCGGCGATCAATCTTTTCAATGAGCTCGGCGAACTTGC
P.indica          61 CGGCTTCCATGGTCTTGCCAGTTCGGCGATCAATCTTCTCGATGAGCTCGGAGAACTTGC
AJ249911_P.indi   61 CGGCTTCCATGGTCTTGCCAGTTCGGCGATCAATCTTCTCGATGAGCTCGGAGAACTTGC
JN211112_S.verm   61 CGGCTTCCATGGTCTTACCCGTTCGGCGATCAATCTTCTCGATGAGCTCGGCAAACTTGC

Isolate_1        121 AGGCAATGTGGGCGGTGTGGCAGTCCAAGACTGGGGCGTAACCAGCACCAATCTGACCAG
Isolate_24       121 AGGCAATGTGGGCGGTGTGGCAGTCCAAGACTGGGGCGTAACCAGCACCAATCTGACCAG
Isolate_46       121 AGGCAATGTGGGCGGTGTGGCAGTCCAAGACTGGGGCGTAACCAGCRCCAATCTGACCAG
Isolate_30       121 AGGCAATGTGGGCGGTGTGGCAGTCCAAGACTGGGGCGTAACCAGCACCAATCTGACCAG
Isolate_34       121 AGGCAATGTGGGCGGTGTGGCAGTCCAAGACTGGGGCGTAACCAGCGCCAATCTGACCAG
Isolate_36       121 AGGCAATGTGGGCGGTGTGGCAGTCCAAGACTGGGGCGTAACCAGCRCCAATCTGACCAG
Isolate_12       121 AGGCAATGTGGGCGGTGTGGCAGTCCAAGACTGGGGCGTAACCAGCACCAATCTGACCAG
P.williamsii     121 AGGCAATGTGGGCGGTGTGGCAGTCCAAGACTGGGGCGTAACCAGCRCCAATCTGACCAG
JN211110_P.will  121 AGGCAATGTGGGCGGTGTGGCAGTCCAAGACTGGGGCGTAACCAGCNCCAATCTGACCAG
P.indica         121 AGGCAATGTGAGCGGTGTGGCAGTCCAAGACTGGGGCGTAACCAGCGCCAATCTGACCGG
AJ249911_P.indi  121 AGGCAATGTGAGCGGTGTGGCAGTCCAAGACTGGGGCGTAACCAGCGCCAATCTGACCGG
JN211112_S.verm  121 AGGCAATGTGGGCGGTGTGGCAATCCAAGACTGGGGCGTAACCAGCGCCAATCTGACCGG

Isolate_1        181 GGTGGTTCAAGACGATGACCTGAGCGTTGAACGAGGCAGCCTCCTTGGCGGGGTCGTTCT
Isolate_24       181 GGTGGTTCAAGACGATGACCTGAGCRTTGAACGAGGCAGCCTCCTTGGCGGGGTCGTTCT
Isolate_46       181 GGTGGTTCAAGACGATGACCTGAGCGTTGAACGAGGCAGCCTCCTTGGCGGGGTCGTTCT
Isolate_30       181 GGTGGTTCAAGACGATGACCTGAGCGTTGAACGAGGCAGCCTCCTTGGCGGGGTCGTTCT
Isolate_34       181 GGTGGTTCAAGACGATGACCTGAGCGTTGAACGAGGCAGCCTCCTTGGCGGGGTCGTTCT
Isolate_36       181 GGTGGTTCAAGACGATGACCTGAGCGTTGAACGAGGCAGCCTCCTTGGCGGGGTCGTTCT
Isolate_12       181 GGTGGTTCAAGACGATGACCTGAGCRTTGAACGAGGCAGCCTCCTTGGCGGGGTCGTTCT
P.williamsii     181 GGTGGTTCAAGACGATGACCTGAGCGTTGAACGARGCAGCCTCCTTGGCGGGGTCGTTCT
JN211110_P.will  181 GGTGGTTCAAGACGATGACCTGAGCGTTGAACGANGCAGCCTCCTTGGCGGGGTCGTTCT
P.indica         181 GGTGGTTCAAGACGATGACCTGAGCGTTGAAMGATGCAGCCTCCTTGGCGGGGTCGTTCT
AJ249911_P.indi  181 GGTGGTTCAAGACGATGACTTGAGCGTTGAAAGATGCAGCCTCCTTGGCGGGGTCGTTCT
JN211112_S.verm  181 GGTGATTTAAGACGATGACCTGAGCGTTGAACGAAGCAGCCTCCTTGGCGGGGTCGTTCT

Isolate_1        241 TCGAGTCCGAGGCGACGTTTCCACGACGGATATCCTTGACCGACACGTTCCTATACAGCA
Isolate_24       241 TCGAGTCCGAGGCGACGTTTCCACGACGGATATCCTTGACCGACACGTTCCTATACAGCA
Isolate_46       241 TCGAGTCCGAGGCGACGTTTCCACGACGGATATCCTTGACCGACACGTTCCTATACAGCA
Isolate_30       241 TCGAGTCCGAGGCGACGTTTCCACGACGGATATCCTTGACCGACACGTTCCTATACAGCA
Isolate_34       241 TCGAGTCCGAGGCGACGTTTCCACGACGGATATCCTTGACCGACACGTTCCTATACAGCA
Isolate_36       241 TCGAGTCYGAGGCGACGTTTCCACGACGGATATCCTTGACCGACACGTTCCTATACAGCA
Isolate_12       241 TCGAGTCCGAGGCGACGTTTCCACGACGGATATCCTTGACCGACACGTTCCTATACAGCA
P.williamsii     241 TCGAGTCYGAGGCGACGTTTCCACGACGGATATCCTTGACCGACACGTTCCTATACAGCA
JN211110_P.will  241 TCGAGTCNGAGGCGACGTTTCCACGACGGATATCCTTGACCGACACGTTCCTATACAGCA
P.indica         241 TCGAGTCCGAAGCGACGTTTCCACGGCGGATATCCTTGACCGACACGTTCCTATACAGCG
AJ249911_P.indi  241 TCGAGTCCGAAGCGACGTCTCCACGGCGGATATCCTTGACCGACACGTTCCTATACAGCG
JN211112_S.verm  241 TCGAGTCGGAGGCGACGTTTCCACGACGGATATCCTTGACCGACACGTT-----------
```

```
Isolate_1         301 ATGAGACGATGAACGACACAACAAAAYAGGGGTAAARACWYACTTGACGTTGAAACCGAC
Isolate_24        301 ATGAGACGATGAACGACACAACAAAACAGGGGTAAARACTCACTTGACGTTGAAACCGAC
Isolate_46        301 ATGAGACGATGAACGACACAACAAAACAGGRGTAAARACTCACTTGACGTTGAAACCGAC
Isolate_30        301 ATGAGACGATGAACGACACAACAAAACAGGGGTAAARACTCACTTGACGTTGAAACCGAC
Isolate_34        301 ATGAGACGATGAACGACACAACAAAACAGGGGTAAAAACTCACTTGACGTTGAAACCGAC
Isolate_36        301 ATGAGACGATGAACGACACAACAAAACAGGGGTAAAAACTCACTTGACGTTGAAACCGAC
Isolate_12        301 ATGAGACGATGAACGACACAACAAAACAGGGGTAAARACTYACTTGACGTTGAAACCGAC
P.williamsii      301 ATGAGACGATGAACGACACAACAAAACAGGGGTAAAGACTCACTTGACGTTGAAACCGAC
JN211110_P.will   301 ATGAGACGATGAACGACACAACAAAACAGGGGTAAAGACTCACTTGACGTTGAAACCGAC
P.indica          301 ATGAGACGATGAACGAGACGACAA---AGAAACGCAAGCTTACTTGACGTTGAARCCGAC
AJ249911_P.indi   301 ATGAGACGATGAACGAGACGACAA---AGAAACGCAAGCTTACTTGACGTTGAAGCCGAC
JN211112_S.verm   290 ------------------------------------------CTTGACGTTGAAGCCGAC

Isolate_1         361 GTTGTCTCCGGGCAAGCCCTCAGAGAGCTGCTCGTGGTGCATTTCGACGGACTTGACTTC
Isolate_24        361 GTTGTCTCCGGGCAAGCCCTCAGAGAGCTGCTCGTGGTGCATTTCGACGGACTTGACTTC
Isolate_46        361 GTTGTCTCCGGGCAAGCCCTCAGAGAGCTGCTCGTGGTGCATTTCGACGGACTTGACTTC
Isolate_30        361 GTTGTCTCCGGGCAAGCCCTCAGAGAGCTGCTCGTGGTGCATTTCGACGGACTTGACTTC
Isolate_34        361 GTTGTCTCCGGGCAAGCCCTCAGAGAGCTGCTCGTGGTGCATTTCGACGGACTTGACTTC
Isolate_36        361 GTTGTCTCCGGGCAAGCCCTCAGAGAGCTGCTCGTGGTGCATTTCGACGGACTTGACTTC
Isolate_12        361 GTTGTCTCCGGGCAAGCCCTCAGAGAGCTGCTCGTGGTGCATTTCGACGGACTTGACTTC
P.williamsii      361 GTTGTCTCCGGGYAAGCCCTCAGAGAGCTGCTCGTGGTGCATTTCGACGGACTTGACTTC
JN211110_P.will   361 GTTGTCTCCGGGNAAGCCCTCAGAGAGCTGCTCGTGGTGCATTTCGACGGACTTGACTTC
P.indica          358 GTTGTCTCCGGGCAAGCCCTCAGCGAGCTGCTCGTGGTGCATTTCGACGGACTTGACTTC
AJ249911_P.indi   358 GTTGTCTCCGGGCAAGCCCTCAGCGAGCTGCTCGTGGTGCATTTCGACGGACTTGACTTC
JN211112_S.verm   308 GTTGTCTCCGGGCAAACCCTCAGTGAGCTGTTCGTGATGCATTTCGACGGACTTGACTTC

Isolate_1         421 AGTGGTGACGTTCGAGGGAGCGAAGGAGACGACCATGCCGGGCTTGATGATACCAGTTTC
Isolate_24        421 AGTGGTGACGTTCGAGGGAGCGAAGGAGACGACCATGCCGGGCTTGATGATACCAGTTTC
Isolate_46        421 AGTGGTGACGTTCGAGGGAGCGAAGGAGACGACCATGCCGGGCTTGATGATACCAGTTTC
Isolate_30        421 AGTGGTGACGTTCGAGGGAGCGAAGGAGACGACCATGCCGGGCTTGATGATACCAGTTTC
Isolate_34        421 AGTGGTGACGTTCGAGGGAGCGAAGGAGACGACCATGCCGGGCTTGATGATACCAGTTTC
Isolate_36        421 AGTGGTGACGTTCGAGGGAGCGAAGGAGACGACCATGCCGGGCTTGATGATACCAGTTTC
Isolate_12        421 AGTGGTGACGTTCGAGGGAGCGAAGGAGACGACCATGCCGGGCTTGATGATACCAGTTTC
P.williamsii      421 AGTGGTGACGTTCGAGGGAGCGAAGGAGACGACCATGCCGGGCTTGATGATACCAGTTTC
JN211110_P.will   421 AGTGGTGACGTTCGAGGGAGCGAAGGAGACGACCATGCCGGGCTTGATGATACCAGTTTC
P.indica          418 AGTGGTGACGTTCGAGGGAGCAAAGGAGACGACCATGCCGGGCTTGATGATACCAGTTTC
AJ249911_P.indi   418 AGTGGTGACGTTCGAGGGAGCAAAGGAGACGACCATGCCGGGCTTGATGATACCAGTTTC
JN211112_S.verm   368 AGTGGTGACGTTGGAGGGAGCAAACGCGACGATCATGCCAGCCTTGATAACACCGGTTTC

Isolate_1         481 GACACGACCGACTGGGACCGTGCCAATACCACCAATCTTGTACACATCCTGGAGTGGGAG
Isolate_24        481 GACACGACCGACTGGGACCGTGCCAATACCACCAATCTTGTACACATCCTGGAGTGGRAG
Isolate_46        481 GACACGACCGACTGGGACCGTGCCAATACCACCAATCTTGTACACATCCTGGAGTGGRAG
Isolate_30        481 GACACGACCGACTGGRACCGTGCCAATACCACCAATCTTGTACACATCCTGGAGTGGGAG
Isolate_34        481 GACACGACCGACTGGGACCGTGCCAATACCACCAATCTTGTACACATCCTGGAGTGGGAG
Isolate_36        481 GACACGACCGACTGGGACCGTGCCAATACCACCAATCTTGTACACATCCTGGAGTGGGAG
Isolate_12        481 GACACGACCGACTGGGACCGTGCCAATACCACCAATCTTGTACACATCCTGGAGTGGGAG
P.williamsii      481 GACACGACCGACTGGGACCGTGCCAATACCACCAATCTTGTACACATCCTGGAGTGGGAG
JN211110_P.will   481 GACACGACCGACTGGGACCGTGCCAATACCACCAATCTTGTACACATCCTGGAGTGGGAG
P.indica          478 GACACGACCGACTGGGACCGTACCAATACCACCAATCTTGTACACATCCTGGAGTGGGAG
AJ249911_P.indi   478 GACACGACCGACTGGGACCGTACCAATACCACCAATCTTGTACACATCCTGGAGTGGGAG
JN211112_S.verm   428 AACACGACCGACGGGCACCGTTCCAATACCACCAATCTTGTACACGTCCTGAAGGGGAG

Isolate_1         541 ACGGAGGGGCTTGTCGGACGGGCGGACKGGGGGTTCAATGGCGTCAATGGCGTCGACGAG
Isolate_24        541 ACGGAGGGGCTTGTCGGACGGGCGGACKGGGGGTTCAATGGCGTCAATGGCGTCGACGAG
Isolate_46        541 ACGGAGGGGCTTGTCGGACGGGCGGACKGGGGGTTCAATGGCGTCAATGGCGTCGACGAG
Isolate_30        541 ACGGAGGGGCTTGTCGGACGGGCGGACKGGGGGTTCAATGGCGTCAATGGCGTCGACGAG
Isolate_34        541 ACGGAGGGGCTTGTCGGACGGGCGGACGGGGGGTTCAATGGCGTCAATGGCGTCGACGAG
Isolate_36        541 ACGGAGGGGCTTGTCGGACGGGCGGACSGGGGGTTCAATGGCGTCAATGGCGTCGACGAG
Isolate_12        541 ACGGAGGGGCTTGTCGGACGGGCGGACTGGGGGTTCAATGGCGTCAATGGCGTCGACGAG
P.williamsii      541 ACGGAGGGGCTTGTCGGACGGGCGGACTGGGGGTTCAATGGCGTCAATGGCGTCGACGAG
JN211110_P.will   541 ACGGAGGGGCTTGTCGGACGGGCGGACTGGGGGTTCAATGGCGTCAATGGCGTCGACGAG
P.indica          538 ACGGAGGGGCTTGTCGGACGGGCGGACTGGGGGTTCGATGGCGTCGATGGCGTCGACGAG
AJ249911_P.indi   538 ACGGAGGGGCTTGTCGGACGGGCGGACTGGGGGTTCGATGGCGTCGATGGCGTCGACGAG
JN211112_S.verm   488 ACGGAGGGGCTTGTCGGAAGGACGGGTTGGGGGTTCGATGGCATCGATGGCGTCGAGGAG
```

```
Isolate_1          601 GGTCTTGCCGGTGGCAGGACTCGACGAGCCCTTGACCTCCTTCGACCATCCCTTGTACCA
Isolate_24         601 GGTCTTGCCGGTGGCAGGACTCGACGAGCCCTTGACCTCCTTCGACCATCCCTTGTACCA
Isolate_46         601 GGTCTTGCCGGTGGCAGGACTCGACGAGCCCTTGACCTCCTTCGACCATCCCTTGTACCA
Isolate_30         601 GGTCTTGCCGGTGGCAGGACTCGACGAGCCCTTGACCTCCTTCGACCATCCCTTGTACCA
Isolate_34         601 GGTCTTGCCGGTGGCAGGACTCGACGAGCCCTTGACCTCCTTCGACCATCCCTTGTACCA
Isolate_36         601 GGTCTTGCCGGTGGCAGGACTCGACGAGCCCTTGACCTCCTTCGACCATCCCTTGTACCA
Isolate_12         601 GGTCTTGCCGGTGGCAGGACTCGACGAGCCCTTGACCTCCTTCGACCATCCCTTGTACCA
P.williamsii       601 GGTCTTGCCGGTGGCAGGACTCGACGAGCCCTTGACCTCCTTCGACCATCCCTTGTACCA
JN211110_P.will    601 GGTCTTGCCGGTGGCAGGACTCGACGAGCCCTTGACCTCCTTCGACCATCCCTTGTACCA
P.indica           598 GGTCTTGCCGGTGGCAGGACTCGACGAGCCCTTGACCTCCTTCGACCATCCCTTGTACCA
AJ249911_P.indi    598 GGTCTTGCCGGTGGCAGGACTCGACGAGCCCTTGACCTCCTTCGACCATCCCTTGTACCA
JN211112_S.verm    548 GGTCTTACCCTTGACGACACC------ACCCTTGCCTTCCTTGGTCCATCCCTTGTACCA

Isolate_1          661 TGGCATGCTATTCGTGCGTTAGACGTGATCGAMA---TAAGCGAGAGAAG-AMYGGCTTA
Isolate_24         661 TGGCATGCTATTCGTGCGTTAGACGTGATCGAMA---TAAGCGAGAGAAG-AACGGCTTA
Isolate_46         661 TGGCATGCTATTCGTGCGTTAGACGTGATCGAMA---TAAGCGAGAGAAG-AACGGCTTA
Isolate_30         661 TGGCATGCTATTCGTGCGTTAGACGTGATCGAMA---TAAGCGAGAGAAG-AACGGCTTA
Isolate_34         661 TGGCATGCTATTCGTGCGTTAGACATGATCGAAA---TAAGCGAGAGAAG-AACGGCTTA
Isolate_36         661 TGGCATGCTATTCGTGCGTTAGACRTGATCGAAA---TAAGCGAGAGAAG-AACGGCTTA
Isolate_12         661 TGGCATGCTATTCGTGCGTTAGACRTGATCRWMR---KAAGCGAGAGAAG-AASGGCTTA
P.williamsii       661 TGGCATGCTATTCGTGCGTTAGACATGATCATAG---GAAGCGAGAGAAG-AAGGGCTTA
JN211110_P.will    661 TGGCATGCTATTCGTGCGTTAGACATGAATCATAGG-AAAGCGAGAGAAGAAAGGGCTTA
P.indica           658 GGGCATGCTATTCGTGCGTCAGATGTAATC-------ACGGGTGAGCGTG-ACCTGCTTA
AJ249911_P.indi    658 GGGCATGCTATTCGTGCGTCAGATGTAATC-------ACGGGTGAGCGTG-ACCTGCTTA
JN211112_S.verm    602 TGGCATGCTATAAGATTGTAAGAAACAAGGATTATCAAAAAGTGGGGAAA-GCACACGTA

Isolate_1          717 CTTGGTAGAGGGCTCAAGCATGTTGTCACCGTGCCAGCCAGAGATGGGGACGAACGCGAC
Isolate_24         717 CTTGGTAGAGGGCTCAAGCATGTTGTCACCGTGCCAGCCAGAGATGGGGACGAACGCGAC
Isolate_46         717 CTTGGTAGAGGGCTCAAGCATGTTGTCACCGTGCCAGCCAGAGATGGGGACGAACGCGAC
Isolate_30         717 CTTGGTAGAGGGCTCAAGCATGTTGTCACCGTGCCAGCCAGAGATGGGGACGAACGCGAC
Isolate_34         717 CTTGGTAGAGGGCTCAAGCATGTTGTCACCGTGCCAGCCAGAGATGGGGACGAACGCGAC
Isolate_36         717 CTTGGTAGAGGGCTCAAGCATGTTGTCACCGTGCCAGCCAGAGATGGGGACGAACGCGAC
Isolate_12         717 CTTGGTAGAGGGCTCAAGCATGTTGTCACCGTGCCAGCCAGAGATGGGGACGAACGCGAC
P.williamsii       717 CTTGGTAGAGGGCTCAAGCATGTTGTCACCGTGCCAGCCAGAGATGGGGACGAAYGCGAC
JN211110_P.will    720 CTTGGTAGAGGNCTCAAGCATGTTGTCNCCGTGCCAGCCAGAGATGGGGACGAANGCGAC
P.indica           710 CTTGGTGGAGGGCTCGAGCATGTTGTCACCGTGCCAGCCAGAGATGGGGACGAAGGCGAC
AJ249911_P.indi    710 CTTGGTGGAGGGCTCGAGCATGTTGTCACCGTGCCAGCCAGAGATGGGGACGAAGGCGAC
JN211112_S.verm    661 CTTGGCAGACTCCTCAATCATGTTGTCACCGTGCCATCCGGAGATGGGGACGAAGGCAAC

Isolate_1          777 CGTCTTGGGGTTGTAKCCGACCTTCTTGATAAAGTTGGAGGTTTCCTTGACGATTTCGTT
Isolate_24         777 CGTCTTGGGGTTGTAACCGACCTTCTTGATAAAGTTGGAGGTTTCCTTGACGATTTCGTT
Isolate_46         777 YGTCTTGGGGTTGTARCCGACCTTCTTGATAAAGTTGGAGGTTTCCTTGACGATTTCGTT
Isolate_30         777 CGTCTTGGGGTTGTARCCGACCTTCTTGATAAAGTTGGAGGTTTCCTTGACGATTTCGTT
Isolate_34         777 CGTCTTGGGGTTGTAACCGACCTTCTTGATAAAGTTGGAGGTTTCCTTGACGATTTCGTT
Isolate_36         777 CGTCTTGGGGTTGTAACCGACCTTCTTGATAAAGTTGGAGGTTTCCTTGACGATTTCGTT
Isolate_12         777 CGTCTTGGGGTTGTARCCGACCTTCTTGATAAAGTTGGAGGTTTCCTTGACGATTTCGTT
P.williamsii       777 CGTCTTGGGGTTGTAGCCGACCTTCTTGATAAAGTTGGAGGTTTCCTTGACGATTTCGTT
JN211110_P.will    780 CGTCTTGGGGTTGTAGCCGACCTTCTTGATAAAGTTGGAGGTTTCCTTGACGATTTCGTT
P.indica           770 CGTCTTGGGGTTGTATCCGACCTTCTTGATGAAGTTGGAGGTTTCCTTGACGATTTCGTT
AJ249911_P.indi    770 CGTCTTGGGGTTGTATCCGACCTTCTTGATGAAGTTGGAGGTTTCCTTGACGATTTCGTT
JN211112_S.verm    721 AGTCTTGGGGTTGTATCCGACCTTCTTGATGAAGTTGGAAGTTTCCTTGATGATTTCATC

Isolate_1          837 GAAGCGCGACTCGGACCACTAGACGCCAAATCGTCAGTTTTGACCAAGGRTACRACNNNN
Isolate_24         837 GAAGCGCGACTCGGACCACTAGACGCCAAATCGTCAGTTTTGACCAAGGGTACRGCGCGR
Isolate_46         837 GAAGCGCGACTCGGACCACTAGACGCCAAATCGTCAGTTTTGACCAAGGGTACAGCGCGR
Isolate_30         837 GAAGCGCGACTCGGACCACTAGACGCCAAATCGTCAGTTTTGACCAAGGRTACARCGCGG
Isolate_34         837 GAAGCGCGACTCGGACCACTAGACGCCAAATCGTCAGTTTTGACCAAGGGTACAGCGCGG
Isolate_36         837 GAAGCGCGACTCGGACCACTAGACGCCAAATCGTCAGTTTTGACCAAGGGTACAGCGCGG
Isolate_12         837 GAAGCGCGACTCGGACCACTAGACGCCAAATCGTCAGTTTTGACCAAGGRTACARCGCGR
P.williamsii       837 GAAGCGCGACTCGGACCACTAGACGCCAAATCGTCAGTTTTGACCAAGGATACAACGCGG
JN211110_P.will    840 GAAGCGCGACTCGGACCACTAGACGCCAAATCGTCAGTTTTGACCAAGGATACAACGCGG
P.indica           830 GAAGCGGGCCTCAGACCACTGGACA--AGATTGTCAGT------CGACGAGARAGTGCAG
AJ249911_P.indi    830 GAAGCGGGCCTCAGACCACTGGACA--AGATTGTCAGT------CGACGAGAGAGTGCAG
JN211112_S.verm    781 GAATCGGCTCTCGGACCACTGTACAAAGAATCA--GGT------TCGGCTCGGCCCGTGT
```

```
                       *
Isolate_1        897 N---------
Isolate_24       897 GG---TTCAA
Isolate_46       897 GG---TTCAA
Isolate_30       897 GG---NNNNN
Isolate_34       897 G----TTCAA
Isolate_36       897 G----NNNNN
Isolate_12       897 GG---NNNNN
P.williamsii     897 GGGAGTTCAA
JN211110_P.will  900 GGGAGTTCAA
P.indica         882 GCGTGCAAGG
AJ249911_P.indi  882 GCGTGCAAGG
JN211112_S.verm  833 GTAACGCGGC
```

Figure S3. Multiple sequence alignments of *Piriformospora ITS* (A) and *TEF1α* (B) sequences. *ITS* and *TEF1α* contig sequences of the Congolese isolates, and of the *P. indica* and *P. williamsii* reference strains used in this study, were assembled in Pregap4 and Gap4 (Bonfield et al., 1995). The new sequences were deposited in GenBank under the accession numbers KY509316-KY509330. In addition, available reference sequences of the four described species within the Serendipitaceae were retrieved from the database: *Piriformospora indica* (KF061284 and AJ249911), *P. williamsii* (JN211110), *S. vermifera* MAFF 305830 (EU626002 and JN211112), and *S. herbamans* (KF061285). Multiple sequence alignments in Clustal format (forward-strand sequences for *ITS* and reverse complement for *TEF1α*) were constructed with MAFFT version 7 using the L-INS-i strategy (Katoh and Standley, 2013), manually refined in ProSeq3 (Filatov, 2009) and further edited with BOXSHADE version 3.21 (Hofmann and Baron, 1992). The presence of two different nucleotides at the same position in the template is specified by IUPAC ambiguity codes. Parts of the sequence that could not be determined because of series of superimposed regions are indicated with the code N. For the *ITS* sequences, this pattern of 'double peaks' is observed for the isolates 1, 24, 30, 46 and *P. williamsii*, and always starts at position 533 in the above *ITS* alignment (indicated with *) or at position 588 when using the *P. indica* GenBank sequence as a reference (KF061284). Because of additional ambiguities in the sequence of *P. williamsii*, the nucleotides between positions 187 and 308 in the *ITS* alignment could also not be determined for this isolate. For the *TEF* alignment, the superimposed regions start around position 893 for isolate 1 or position 902 for isolates 12, 30 and 36 (indicated with *), which corresponds to position 2,355 of the *P. indica* GenBank reference sequence (AJ249911). This is situated in intron number 5.

The alignments show that the seven selected Congolese isolates (as well as the sequence retrieved from a chlamydospore-containing maize root of trap culture D, highlighted in yellow in the *ITS* alignment) belong to the *Piriformospora* cluster of the Serendipitaceae. In addition, it is clear that the new isolates are more closely related to *P. williamsii* than to *P. indica* which argues in favor of a *Piriformospora* '*williamsii*' species complex.

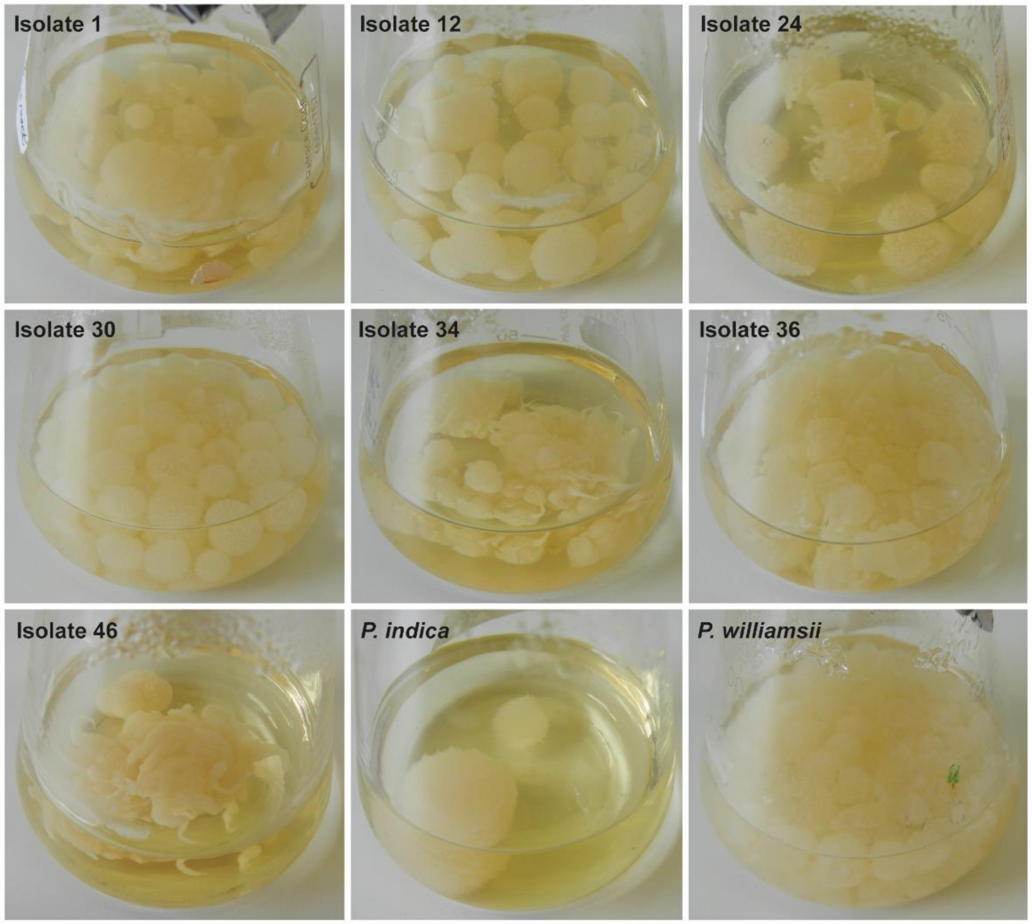

Figure S4. *Piriformospora* mycelium in CM liquid culture. *Piriformospora* isolates 1, 12, 24, 30, 34, 36 and 46, and *P. indica* and *P. williamsii*, were cultured in 100-ml Erlenmeyer flasks containing 50 ml CM liquid medium at 27°C and under continuous shaking (120 rpm) for eighteen days. Mycelium was typically conglomerated to small globose balls.

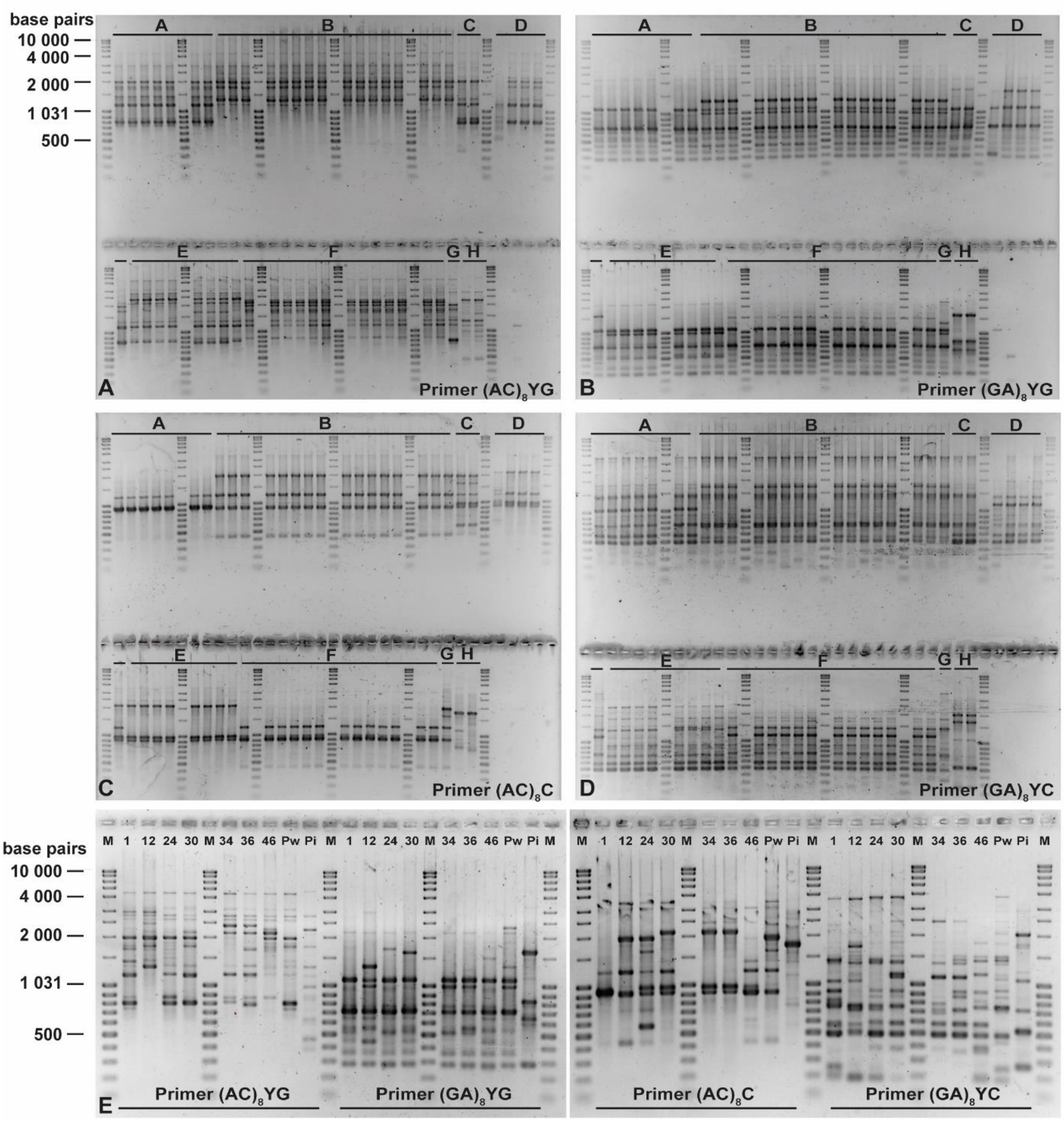

Figure S5. ISSR fingerprinting profiles of *Piriformospora* isolates. (**A-D**) ISSR banding profiles of all 51 Congolese isolates, and *P. indica* and *P. williamsii*, for the primers $(AC)_8YG$ (**A**), $(GA)_8YG$ (**B**), $(AC)_8C$ (**C**) and $(GA)_8YC$ (**D**). A, isolates 1-7 (MAS 2); B, isolates 8-23 (SS 3); C, isolates 24-25 (MAN 2); D, isolates 26-30 (MAN 3); E, isolates 31-38 (MAS 6); F, isolates 39-51 (MAS 9); G, *P. williamsii*; H, *P. indica* (two different DNA extractions). (**E**) ISSR banding profiles of the selected subset of Congolese isolates (1, 12, 24, 30, 34, 36 and 46), and *P. indica* (Pi) and *P. williamsii* (Pw), generated by the same four primers. Banding patterns were identical to the profiles generated in the first analysis (A-D), demonstrating the reproducibility across time. The 1.5% gels were run at 100 V for 2.5 h and visualized with ethidium bromide. The MassRuler DNA Ladder Mix (Thermo Fisher Scientific) was used as a marker.

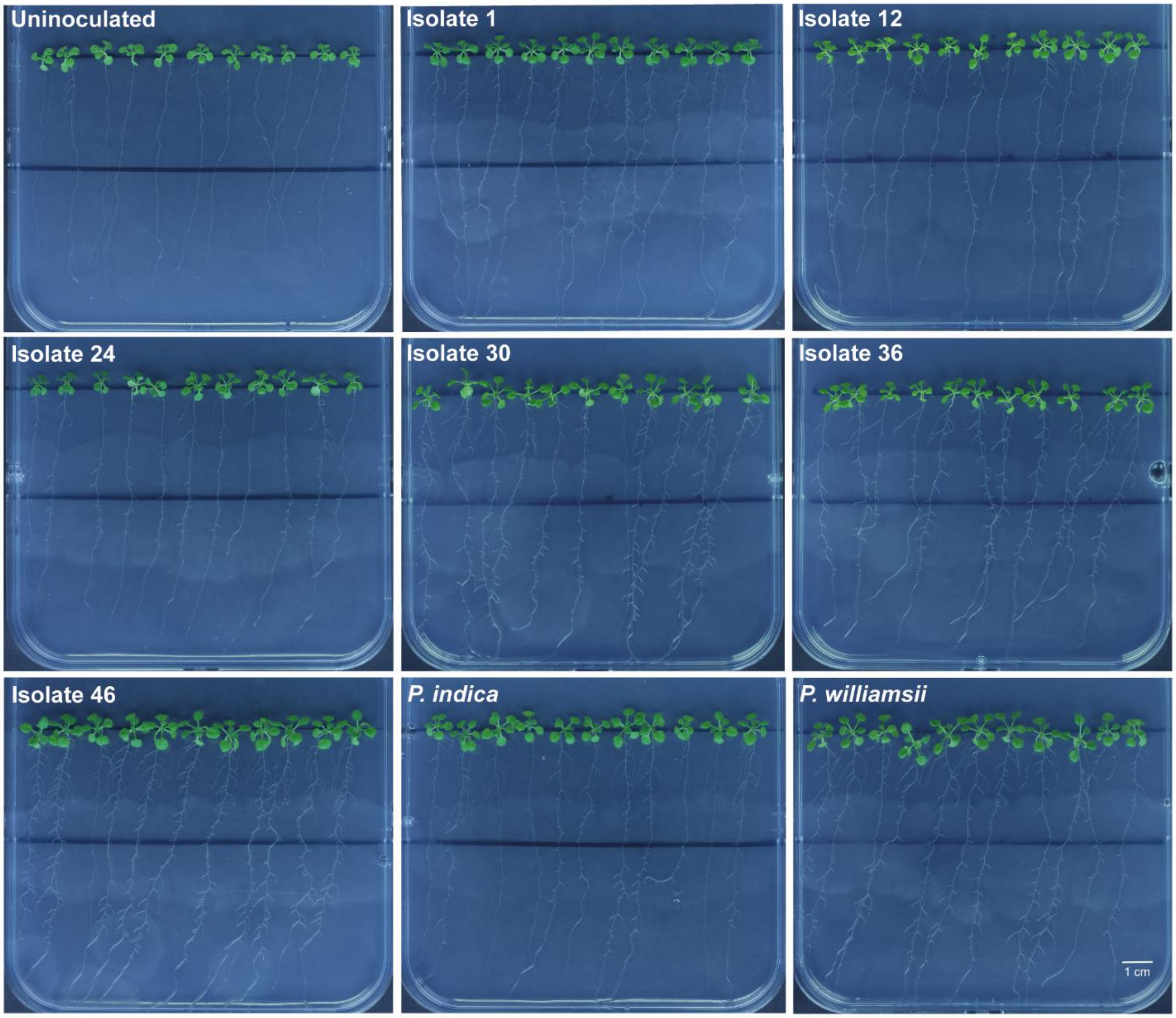

Figure S6. Growth promotion effect of *Piriformospora* isolates on *Arabidopsis thaliana* (Col-0) in an *in vitro* assay. Five-day-old *Arabidopsis* seedlings growing on half-strength MS medium without sucrose were inoculated with 30,000 spores of the selected subset of Congolese isolates (1, 12, 24, 30, 34, 36 and 46), and *P. indica* and *P. williamsii*, at a distance of 3.5 cm below the seedlings (black line). Shoot fresh weight was positively affected by *Piriformospora* inoculation. A pronounced stimulation of lateral root formation and lateral root growth in the mature zone was also observed. More detailed close-ups are included in Figure 7E.

References

Bayraktar, H., Dolar, F.S., and Maden, S. (2008). Use of RAPD and ISSR Markers in Detection of Genetic Variation and Population Structure among *Fusarium oxysporum* f. sp. *ciceris* Isolates on Chickpea in Turkey. *J Phytopathol* 156, 146-154. doi: 10.1111/j.1439-0434.2007.01319.x

Bonfield, J.K., Smith, K.f., and Staden, R. (1995). A new DNA sequence assembly program. *Nucleic Acids Res* 23, 4992-4999.

Boss, D., Maurhofer, M., Zala, M., Défago, G., and Brunner, P.C. (2007). ISSR fingerprinting for the assessment of the bindweed biocontrol agent *Stagonospora convolvuli* LA39 after field release. *Lett Appl Microbiol* 45, 244-251. doi: 10.1111/j.1472-765X.2007.02177.x

Chadha, S., and Gopalakrishna, T. (2009). Informativeness of dinucleotide repeat-based primers in fungal pathogen of rice *Magnaporthe grisea*. *Microbiol Res* 164, 276-281. doi: 10.1016/j.micres.2006.11.019

Consolo, V.F., Ortega, L.M., Salerno, G., Astoreca, A.L., and Alconada, T.M. (2015). Genetic diversity of *Fusarium graminearum sensu lato* isolates from wheat associated with Fusarium Head Blight in diverse geographic locations of Argentina. *Rev Argent Microbiol* 47, 245-250. doi: 10.1016/j.ram.2015.05.004

Curlevski, N.J., Chambers, S.M., Anderson, I.C., and Cairney, J.W. (2009). Identical genotypes of an ericoid mycorrhiza-forming fungus occur in roots of *Epacris pulchella* (*Ericaceae*) and *Leptospermum polygalifolium* (*Myrtaceae*) in an Australian sclerophyll forest. *FEMS Microbiol Ecol* 67, 411-420. doi: 10.1111/j.1574-6941.2008.00637.x.

Estrada, M.E., Camacho, M.V., and Benito, C. (2007). The molecular diversity of different isolates of *Beauveria bassiana* (Bals.) Vuill. as assessed using intermicrosatellites (ISSRs). *Cell Mol Biol Lett* 12, 240-252. doi: 10.2478/s11658-006-0069-4

Filatov, D.A. (2009). Processing and population genetic analysis of multigenic datasets with ProSeq3 software. *Bioinformatics* 25, 3189-3190. doi: 10.1093/bioinformatics/btp572

Gardes, M., and Bruns, T.D. (1993). ITS primers with enhanced specificity for basidiomycetes-application to the identification of mycorrhizae and rusts. *Mol Ecol* 2, 113-118. doi: 10.1111/j.1365-294X.1993.tb00005.x

Hofmann, K., and Baron, M.D. (1992). *Boxshade server.* Swiss Institute of Bioinformatics. Available online at: http://www.ch.embnet.org/software/BOX_form.html

Johnson, C.M., Stout, P.R., Broyer, R.C., and Carlton, A.B. (1957). Comparative chlorine requirements of different plant species. *Plant Soil* 8, 337-353. doi:10.1007/BF01666323

Katoh, K., and Standley, D.M. (2013). MAFFT Multiple Sequence Alignment Software Version 7: Improvements in Performance and Usability. *Mol Biol Evol* 30, 772-780. doi: 10.1093/molbev/mst010

Martin, K.J., and Rygiewicz, P.T. (2005). Fungal-specific PCR primers developed for analysis of the ITS region of environmental DNA extracts. *BMC Microbiol* 5:28. doi: 10.1186/1471-2180-5-28

O'Donnell, K. (1993). "*Fusarium* and its near relatives," in *The Fungal Holomorph: Mitotic, Meiotic and Pleomorphic Speciation in Fungal Systematics*, eds. D.R. Reynolds, and J.W. Taylor (Wallingford: CAB International), 225-233.

Palmero, D., Rubio-Moraga, A., Galvez-Patón, L., Nogueras, J., Abato, C., Gómez-Gómez, L. et al. (2014). Pathogenicity and genetic diversity of *Fusarium oxysporum* isolates from corms of *Crocus sativus*. *Ind Crops Prod* 61, 186-192. doi: 10.1016/j.indcrop.2014.06.051

Pham, G.H., Kumari, R., Singh, A., Sachdev, M., Prasad, R., Kaldorf, M. et al. (2004). "Axenic cultures of *Piriformospora indica*," in *Plant Surface Microbiology*, eds. A. Varma, L. Abbott, D. Werner, and R. Hampp (Berlin: Springer-Verlag), 593-616.

Pintye, A., Bereczky, Z., Kovács, G.M., Nagy, L.G., Xu, X., Legler, S.E. et al. (2012). No indication of strict host associations in a widespread mycoparasite: grapevine powdery mildew (*Erysiphe necator*) is attacked by phylogenetically distant *Ampelomyces* strains in the field. *Phytopathology* 102, 707-716. doi: 10.1094/PHYTO-10-11-0270

White, T.J., Bruns, T., Lee, S., and Taylor, J.W. (1990). "Amplification and direct sequencing of fungal ribosomal RNA genes for phylogenetics," in *PCR protocols: a guide to methods and applications*, eds. M.A. Innis, D.H. Gelfand, J.J. Sninsky, and T.J. White (New York: Academic Press), 315-322.
